# The Epithelial and Stromal Immune Microenvironment in Gastric Cancer: A Comprehensive Analysis Reveals Prognostic Factors with Digital Cytometry

**DOI:** 10.3390/cancers13215382

**Published:** 2021-10-27

**Authors:** Wenjun Shen, Guoyun Wang, Georgia R. Cooper, Yuming Jiang, Xin Zhou

**Affiliations:** 1 Department of Bioinformatics, Shantou University Medical College, Shantou 515041, China; 18gywang@stu.edu.cn; 2 Stanford Center for Biomedical Informatics Research (BMIR), Department of Medicine, Stanford University, Stanford, CA 94035, USA; 3 Guangdong Provincial Key Laboratory of Infectious Diseases and Molecular Immunopathology, Shantou 515041, China; 4 Department of Biomedical Engineering, Vanderbilt University, Nashville, TN 37235, USA; georgia.r.cooper@vanderbilt.edu (G.R.C.); maizie.zhou@vanderbilt.edu (X.Z.); 5 Department of Radiation Oncology, Stanford University School of Medicine, Stanford, CA 94035, USA; 6 Department of Computer Science, Vanderbilt University, Nashville, TN 37235, USA

**Keywords:** gastric cancer, tumor microenvironment, scRNA-seq, cell mixture deconvolution

## Abstract

**Simple Summary:**

We investigated the tumor microenvironment of gastric cancer (GC) by combining single cell and bulk transcriptomic profiles. We built a novel signature matrix to dissect epithelium and stroma signals from tissue samples using a scRNA-seq data set for GC and then applied cell mixture deconvolution to estimate diverse epithelial, stromal, and immune cell proportions from bulk transcriptome data in four independent GC cohorts. Using a robust computational pipeline, we identified an early malignant epithelial cell (EMEC) population whose proportions were significantly higher in patients with stage I cancer than other stages, and it was predominantly present in tumor samples but not typically found in normal samples. By using univariate and multivariate analyses in the training cohort, we identified that the ratio of EMECs to stromal cells and the ratio of adaptive T cells to monocytes were the most significant prognostic factors within the non-immune and immune factors, respectively. The STEM score, which unifies these two prognostic factors, was an independent prognostic factor of overall survival for GC.

**Abstract:**

Gastric cancer (GC) is the third leading cause of cancer-related deaths worldwide. Tumor heterogeneity continues to confound researchers’ understanding of tumor growth and the development of an effective therapy. Digital cytometry allows interpretation of heterogeneous bulk tissue transcriptomes at the cellular level. We built a novel signature matrix to dissect epithelium and stroma signals using a scRNA-seq data set (GSE134520) for GC and then applied cell mixture deconvolution to estimate diverse epithelial, stromal, and immune cell proportions from bulk transcriptome data in four independent GC cohorts (GSE62254, GSE15459, GSE84437, and TCGA-STAD) from the GEO and TCGA databases. Robust computational methods were applied to identify strong prognostic factors for GC. We identified an EMEC population whose proportions were significantly higher in patients with stage I cancer than other stages, and it was predominantly present in tumor samples but not typically found in normal samples. We found that the ratio of EMECs to stromal cells and the ratio of adaptive T cells to monocytes were the most significant prognostic factors within the non-immune and immune factors, respectively. The STEM score, which unifies these two prognostic factors, was an independent prognostic factor of overall survival (HR = 0.92, 95% CI = 0.89–0.94, $p=2.05\times 10^{-9}$). The entire GC cohort was stratified into three risk groups (high-, moderate-, and low-risk), which yielded incremental survival times (p<0.0001). For stage III disease, patients in the moderate- and low-risk groups experienced better survival benefits from radiation therapy ((HR = 0.16, 95% CI = 0.06–0.4, p<0.0001), whereas those in the high-risk group did not (HR = 0.49, 95% CI = 0.14–1.72, p=0.25). We concluded that the STEM score is a promising prognostic factor for gastric cancer.

## 1. Introduction

Gastric cancer (GC) is a complex and heterogeneous disease from morphological, molecular, and cellular standpoints [1]. Such tumor heterogeneity has been demonstrated in numerous histological and molecular classifications. The Lauren classification separates gastric adenocarcinomas into intestinal, diffuse, and mixed subtypes, which were found to be associated with varying stomach cancer risks [2,3]. The Asian Cancer Research Group (ACRG) classified GC into four molecular subtypes that, based on gene expression data, were associated with distinct molecular alterations, disease progression, and survival outcomes [4]. These subtypes are epithelial-to-mesenchymal transition (EMT), MSS/TP53-, MSS/TP53+, and microsatellite instability (MSI). More recently, The Cancer Genome Atlas (TCGA) research network characterized GC into four genomic subtypes by integrating data from six molecular platforms: array-based somatic copy number analysis, whole-exome sequencing, array-based DNA methylation profiling, messenger RNA sequencing, microRNA (miRNA) sequencing, and reverse-phase protein array, as well as Microsatellite instability (MSI) testing [5]. These genomic subtypes are EBV-positivity (EBV), MSI-high status (MSI), genomically stable (GS), and those exhibiting chromosomal instability (CIN). Each subtype displays distinct molecular and genomic patterns.

Tumor heterogeneity, including the results from the tumor microenvironment (TME), continues to confound researchers’ understanding of tumor growth and the development of an effective therapy [6,7]. Tumors are complex ecosystems that are affected by numerous stromal and immune factors, which dampen or enhance the effects of genetic epithelial alterations [8,9,10,11]. The TME is comprised of tumor cells, tumor stromal cells, endothelial cells, immune cells, and the non-cellular components of extracellular matrix proteins [12,13]. Some essential components of the TME, including cancer-associated fibroblasts (CAF) [14,15], tumor-infiltrating lymphocytes (TIL) [16,17], tumor-associated macrophages (TAM) [18], and other cellular components [19,20,21], have been evaluated to help researchers better understand the role of the TME in gastric cancer risk. Most of these studies have focused on the subsets of cellular components of TME: typically, the stromal and immune cell populations. However, the prognostic values of diverse gastric epithelial cell types in GC risk were still unclear. To our knowledge, a systematic analysis of the prognostic value of diverse epithelial cell types, including cancer cells, MSC, PMC and PC, emerged in early gastric cancer for predicting survival has not been described.

Digital cytometry enables the examination of heterogeneous bulk tissue transcriptomes at the cellular level in addition to using computational methods to quantify cell type composition. In recent years, single-cell RNA-sequencing (scRNA-seq) techniques have offered new insights into tissue samples at the resolution of single cells. The availability of single cell transcriptomic profiles, which are used to build cell-type-specific signature matrices, promote the development of statistical deconvolution methods for estimating cell type compositions in heterogeneous mixture samples [22,23].

This study attempted to investigate the TME of GC with comprehensive epithelial, stromal, and immune cell profiling by combining single-cell and bulk expression profiles. We undertook a comprehensive analysis on 10 non-immune and 7 immune cell populations of the TME of GC, evaluated the prognostic role of a novel TME signature score called STEM score in four independent GC cohorts, and stratified the GC patients into three TME subtypes based on the abundance of four STEM populations (i.e., Stromal cell, adaptive T cell, EMEC, and Monocyte).

## 2. Materials and Methods

### 2.1. Gastric Cancer Bulk Gene Expression Data

We collected four GC gene expression datasets with the associated clinical, pathological, and outcome data: GSE62254 (ACRG), GSE15459, GSE84437, and TCGA-STAD (stomach adenocarcinoma) (Table S1). The ACRG and GSE15459 data sets contained gene expression profiles of 300 and 192 patients, respectively. The raw data (CEL files) of these two data sets were downloaded from the Gene Expression Omnibus (GEO, www.ncbi.nlm.nih.gov/geo/, accessed on 1 September 2021). The CEL files were MAS5-normalized in the R environment using the affy software package. Both data sets were converted to gene-specific expression matrices using the R package hgu133plus2.db. For the GSE84437 data set, we directly downloaded its expression matrix after using quantile normalization from GEO. The R package illuminaHumanv3.db was used to translate probe identifications (IDs) to gene symbols. When multiple probes were present for one gene, we selected the probe with the highest average expression across the samples. The TCGA-STAD data set from The Cancer Genome Atlas (TCGA) was downloaded by using the getTCGA function of R package TCGA2STAT. RNA-Seq version 2 data were processed using RSEM algorithm [24], which generated estimated counts as a proxy for RNA expression levels.

The corresponding clinical, pathological, and outcome data of these data sets were collected as follows. We collected clinical data of the ACRG cohort from the Supplementary Materials of the original publication [4]. For the GSE15459 and GSE84437 cohorts, clinical data were retrieved from the GEO database. We used the getTCGA function of R package TCGA2STAT to obtain clinical and OS data for the TCGA-STAD cohort.

### 2.2. Identification of Ten Non-Immune Cell Populations from Single-Cell RNA-Seq of Gastric Antral Mucosa Biopsies

The single-cell RNA-seq data of patients with gastric premalignant lesions and early GC were downloaded from the GEO database with accession number GSE134520. This data set, profiled by 10× Chromium v2 (3’ assay), consists of 32,332 cells from nine patients with non-atrophic gastritis (NAG), chronic atrophic gastritis (CAG), intestinal metaplasia (IM), and early gastric cancer (EGC). We only included non-immune cell types with more than 20 cells’ sequencing data available at two or more stages for further analysis. During quality control, we further excluded cells with fewer than 500 expressed genes and removed genes detected in less than 2% cells, leaving 9926 genes in a total of 24,874 single cells.

**Figure 1:**
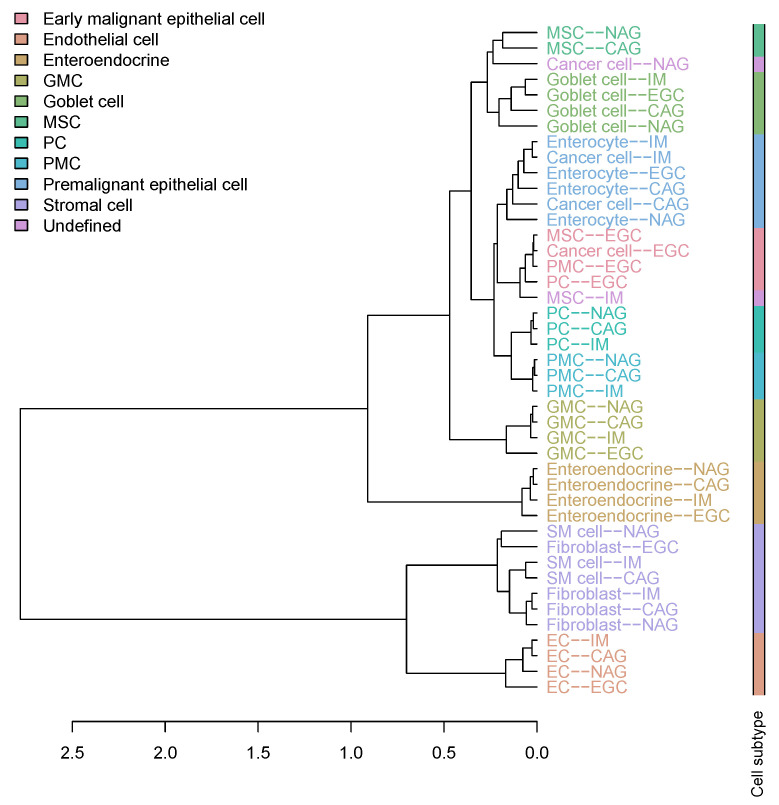
Cluster tree of 11 non-immune cell types of patients with NAG, CAG, IM, and EGC (top 100 genes).

**Figure 2:**
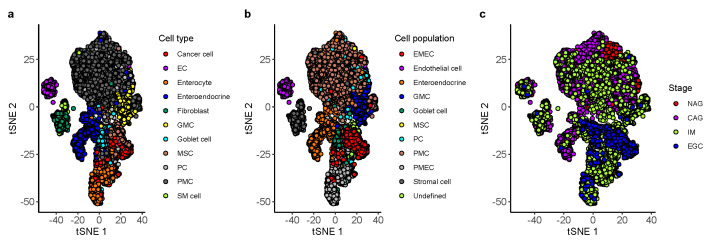
The t-SNE plot of whole transcriptomes of 24,874 cells from gastric antral mucosa biopsies. (**a**) The 11 cell types are denoted by distinct colors. (**b**) The 10 identified cell populations are denoted by distinct colors. (**c**) The 4 stages are denoted by distinct colors.

To explore the hierarchical relationship among cell types in the cascade from gastritis to EGC, agglomerative hierarchical clustering was performed on the gastric scRNA-seq data set. The counts of single-cell gene expression data were summarized across all cells mapped to the same cell type and subject. We then normalized the count summarization matrix by the transcripts per million (TPM) method. Next, we used the two-way analysis of variance (ANOVA) *F* test to identify cell type specific expressed genes. The main effects of cell type and subject were analyzed. The ANOVA analysis tested for differentially expressed genes between a cell type and all other cells. The cell-type-specific expressed genes were identified as the top *N* genes with high a *F* value of cell type compared to the *F* value of the subject. We varied *N* from 50 to 200 with step 50 and found that the clustering result is less sensitive to the value of *N*. Assignment of data points to cluster goblet cell does not change when N∈{50,100}. Assignment of data points to cluster premalignant epithelial cell (PMEC) does not change when N∈{100,150,200}. Assignment of data points to other clusters does not change when varying the value of *N* (Figure 1 and Figures S1–S3). Therefore, we selected the top N=100 genes with high *F* values of cell type compared to *F* values of subjects as the cell-type-specific expressed genes. We next computed the mean expression for the cell-type-specific expressed genes across expression profiles mapped to the same cell type and stage. The hierarchical cluster tree was generated using the Pearson correlation coefficient ($\frac{1-r}{2}$) as the pairwise distance on the log-transformed mean expression profiles and Ward’s linkage distance as the cluster distance. Ten non-immune cell populations consisting of EMEC, PMEC, enteroendocrine, GMC, goblet cell, MSC, PC, PMC, endothelial cell, and stromal cell were identified from the cluster tree (Figure 1). The mapping of 11 non-immune cell types of patients with NAG, CAG, IM, and EGC to 10 cell populations is provided in Table S2 and Figure 2.

### 2.3. Marker Selection and Signature Matrix Construction

To estimate the proportions of non-immune cell populations in the gastric samples, we created a signature matrix composed of the characteristic expression profiles for each of the 10 non-immune cell populations. This signature matrix distinguished EMEC, PMEC, enteroendocrine, GMC, goblet cell, MSC, PC, PMC, endothelial cell, and stromal cell populations. The matrix was generated based on the gastric single cell data. The counts of single cell gene expression data were summarized across all cells mapped to the same cell type and subject then normalized to the count summarization matrix by the TPM method. The expression profiles were averaged within each cell population. This generated a matrix of genes × cell populations. The signature matrix was defined as the sub-matrix formed by a set of cell population-specific marker genes. The marker genes were identified by first selecting genes with a two-fold or higher over differential expressed between one cell population and all other cell populations, then filtering out non-significant genes with a *p*-value larger than 0.01 (one-way ANOVA analysis tested for each gene between a cell population and all other cells). Next, we ranked genes in decreasing order by their fold changes and selected the top *M* genes for each cell population as marker genes. To find the optimal *M*, we constructed an artificial bulk RNA-seq data by summing the scRNA-seq read counts across cells for each biopsy from the gastric scRNA-seq data set. In this case, true cell type proportions are known, which allows the evaluation of deconvolution accuracy. We then created multiple signature matrices by varying *M* from 50 to 250 with step 50 to estimate cell proportions of the artificial bulk RNA-seq data. We found that M=150 achieved the best accuracy (Table S3). Thus, we selected the top M=150 genes for each cell population, which resulted in a signature matrix of 1319 genes by 10 cell populations, named GC10. The signature matrix GC10 and gene signatures for each non-immune cell population are available on Github (https://github.com/wenjshen/STEMscore, accessed on 1 September 2021).

To quantify the proportions of immune cell populations, we used a signature matrix provided in [25] consisting of 1296 genes in 17 immune cell types whose transcriptomic profiling was sorted by RNA-seq. This signature matrix covers the majority of cells that constitute a PBMC sample. We then merged these 17 immune cell types into 7 major lineages according to their biological similarity, resulting in 7 immune cell populations: adaptive T cells, innate T cells, adaptive B cells, natural killer cells, monocytes, dendritic cells, and granulocytes (Table S4).

### 2.4. Deconvolving Bulk Gene Expression Samples

For the deconvolution of the bulk RNA-seq data, the expression profiles of bulk samples were normalized for sequencing depth and gene length using the Transcripts Per Million (TPM) method [26]. We performed deconvolution with support vector regression using the CIBERSORT algorithm [27].

The microarray data were quantile normalized. We next used the CIBERSORTx method [23] to deconvolve bulk samples. Additionally, batch correction was applied to reduce cross-platform variance.

**Figure 3:**
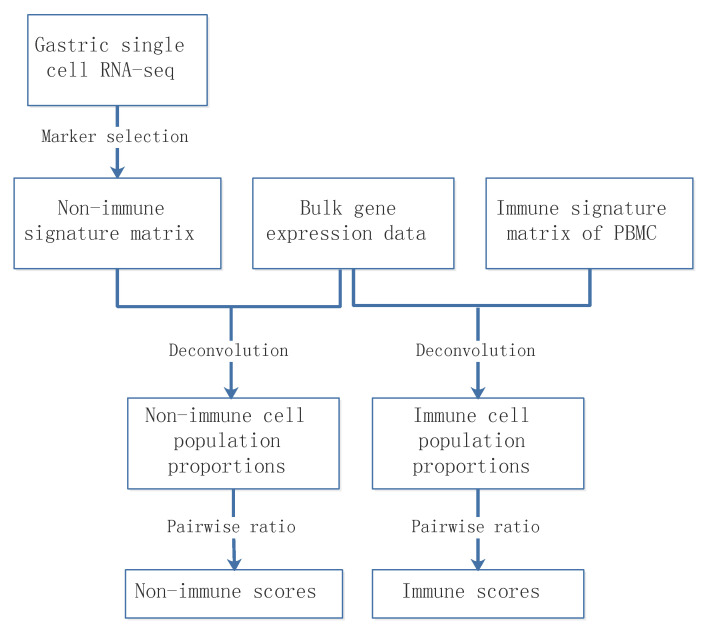
Schematic of the deconvolution of heterogeneous tissue samples.

The deconvolution method was combined with the signature matrices of non-immune and immune cell populations, respectively, to estimate non-immune and immune cell relative fractions of GC samples. CIBERSORT and CIBERSORTx were run with non-negativity and sum-to-1 constraints. Thus, they estimated relative but not absolute fractions of cell populations within a sample, and the values may not be comparable across samples. We therefore defined non-immune scores (or immune scores) by calculating the ratios of relative fractions in each pair of non-immune (or immune) cell populations. The non-immune or immune scores are therefore comparable across samples (Figure 3).

### 2.5. Go Enrichment Analyses

The functional classification of the EMEC gene signatures was identified using Reactome and Gene Ontology (GO) enrichment analyses. Reactome enrichment analyses were performed with the enrichPathway function in the R package ReactomePA (version 1.9.4). Significance in all Reactome enrichment analyses were based on BH-corrected *p* value < 0.15 and the gene counts ≥2. The R package clusterProfiler [28] (version 3.18.1) was used to identify and visualize enriched GO terms. Significance in all GO enrichment analyses was based on BH-corrected *p* value < 0.1 and the gene counts ≥2.

### 2.6. Determination of Optimal Stem Score Cutoff

To identify the statistically optimal cutoff of the STEM score, we analyzed the influence of the STEM score on survival outcome in the ACRG cohort by using univariate Cox proportional hazard modeling. The analysis was performed for a dense set of quantiles from 0.1th to 0.9th, with a 0.01 step. Each analysis divided the entire cohort into two groups, and the cutoff that minimized the *p*-value for testing the risk difference between the two groups was selected.

### 2.7. Tme Subtypes Identification in GC

Each GC sample was represented by four input features of the estimated relative abundance of the cell populations: EMECs, stromal cells, adaptive T cells, and monocytes. Spectral clustering was performed on the meta-cohort (ACRG, GSE15459, GSE84437, and TCGA-STAD) by using the radial basis kernel function to measure the similarity between two samples, which was implemented in the R kernlab package. The optimal number of clusters was chosen using the NbClust function, which was implemented in the R NbClust [29] package. NbClust utilizes 26 different cluster validity indices with Euclidean as the distance measurement method and Ward’s hierarchical clustering as the clustering method to generate a majority rules number of clusters for the GC data set.

### 2.8. Statistical Analysis

All the statistical analyses were performed in the R environment (version 4.0.3). Cumulative survival time was calculated using the Kaplan–Meier method, and the differences in survival curves were analyzed using the log-rank test from R package survminer. Univariate and multivariate analyses were conducted using the Cox proportional hazards regression modeling using the R package survival. For all tests, the *p*-value cutoff for statistical significance was set as 0.05 as the default unless an alternative value was specified. Statistical significance between tumor samples and adjacent normal samples was assessed using Student’s *t* test and indicated as follows: ${}^{*}\;p<0.05,{\;}^{**}\;p<0.01,{\;}^{***}\;p<0.001$ and ${}^{****}\;p<0.0001$.

## 3. Results

### 3.1. Building a Non-Immune Signature Matrix for GC from a Single-Cell Rna-Seq Data Set

We carried out a systematic cell subtype analysis on a single-cell RNA-seq data set of patients with gastric premalignant lesions and early gastric cancer [30]. An unsupervised hierarchical clustering analysis was used to investigate the possibility of identifying different non-immune cell populations in the gastric scRNA-seq data set based on their expression profiles. Hierarchical clustering of the cell types in the cascade stages from non-atrophic gastritis (NAG), chronic atrophic gastritis (CAG), intestinal metaplasia (IM), and early gastric cancer (EGC) in the single cell reference revealed that 24,874 non-immune cells fell into three large groups: epithelial cell types (including cancer cell, enterocyte, enteroendocrine, GMC, goblet cell, MSC, PC, and PMC), stromal cell types (including Fibroblast and SM cells), and endothelial cell types (Figure 1). Notably, the enterocytes and cancer cells that emerged in the CAG and IM biopsies were clustered together. We labeled this cluster as the premalignant epithelial cell (PMEC) population. We also detected a cell population in which cells were clustered by stage instead of by cell type. This cell population consisted of four gastric epithelial cell types (including cancer cells, MSC, PC, and PMC) that emerged uniformly in the EGC biopsy. Therefore, we called it the early malignant epithelial cell (EMEC) subtype. The other eight cell populations were found to be clustered by cell type. Overall, ten cell populations of EMEC, PMEC, enteroendocrine, GMC, goblet cells, MSC, PC, PMC, endothelial cell, and stromal cells were detected in the cluster tree (Figure 1, Table S2). To estimate the proportions of non-immune cell compositions in the gastric cancer samples, we created a cell population-specific signature matrix to distinguish these 10 cell populations. The cell population-specific marker genes were selected by using two-way ANOVA (see Section 2 for details).

### 3.2. Dissecting Epithelium-Stroma-Immune Signals from Gastric Cancer Samples

**Figure 4:**
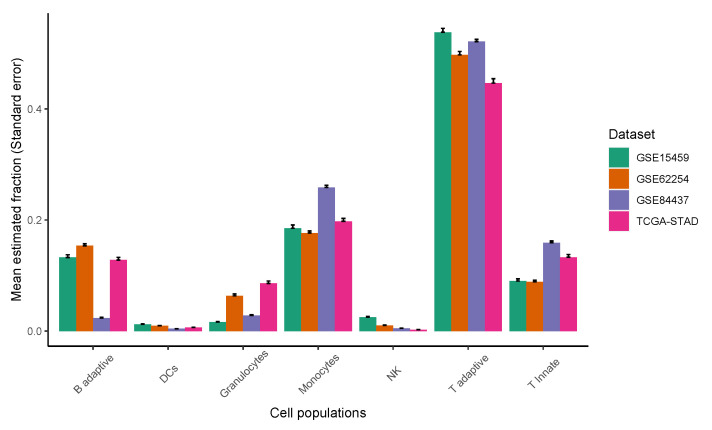
The relative abundance of major classes of immune cell populations for four cohorts of gastric cancer samples.

**Figure 5:**
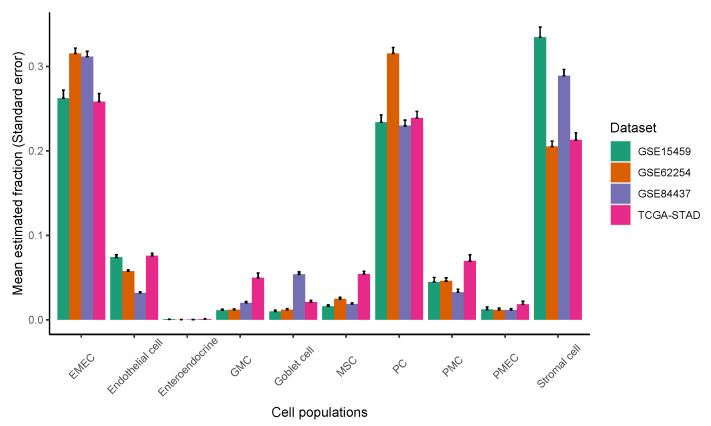
The relative abundance of major classes of non-immune cell populations for four cohorts of gastric cancer samples.

Epithelial, stromal, and immune cell populations comprise the vast majority of gastric tumor cellularity. In order to accurately dissect epithelium-stroma-immune signals from GC samples, we applied CIBERSORT [27] or CIBERSORTx [23] to RNA profiles of GC samples. Our methodology involved two separate deconvolution procedures: an immune cell deconvolution procedure and a non-immune cell deconvolution procedure, which dissect immune and non-immune signals from GC samples, respectively. The immune system is an important determinant of the TME, and we applied CIBERSORT/CIBERSORTx to infer relative immune cell subtype fractions in four cohorts of bulk GC samples by using a signature matrix derived from the peripheral blood mononuclear cell (PBMC) samples (Figure 4, see Section 2 for details). We observed that the T adaptive cell population, comprised of naive and memory CD4 and CD8 T cells, was consistently dominant across the four GC cohorts, followed by the monocyte, B adaptive, and T innate subsets. The single-cell gene expression data sets of GC offer new insights to investigating the GC TME at the resolution of single cells. To enumerate GC non-immune cell proportions, we further applied CIBERSORT/CIBERSORTx by using the single-cell reference profiles to distinguish epithelial, stromal, and endothelial cell subsets in the bulk GC samples (Figure 5, see Section 2 for details). We observed that the EMEC, PC, and stromal cell subsets were highly abundant, while the endothelial cell subset was less abundant in all four cohorts.

**Figure 6:**
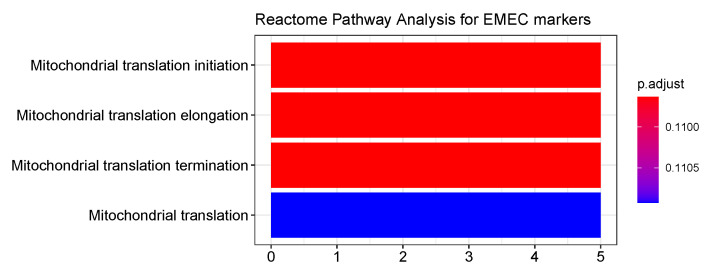
Top Reactome ontologies enriched for the EMEC gene signatures.

The EMEC population is composed of gastric epithelial cells (including cancer cells, MSCs, PCs, and PMCs) that were isolated from early GC patients. We analyzed the functional annotation of the EMEC gene signatures using Reactome and Gene Ontology (GO) enrichment analyses. The EMEC gene signatures were mostly enriched for mitochondrial translation, mitochondrial translational elongation, and mitochondrial translational initiation and termination in the Reactome (Figure 6) and biological process (BP) ontologies (Figure S4a); they were mainly enriched in the mitochondrial inner membrane and mitochondrial ribosome in the cellular component (CC) ontology (Figure S4b). The results suggest that the mitochondria could serve as a GC biomarker for early detection, which is consistent with previous reports showing a role for mitochondria [31,32,33] in the early detection of solid tumors.

**Figure 7:**
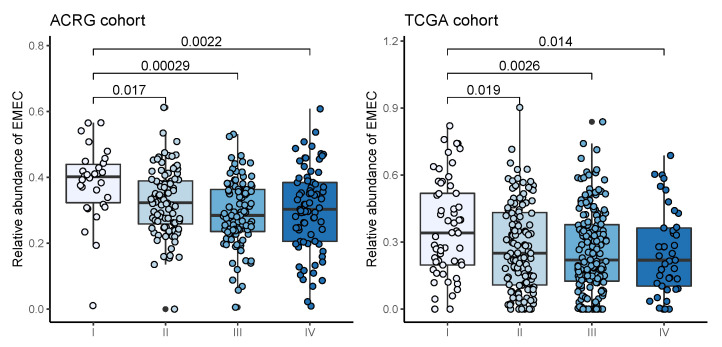
Comparison of the relative abundance of EMEC population across four stages in the ACRG and TCGA cohorts.

**Figure 8:**
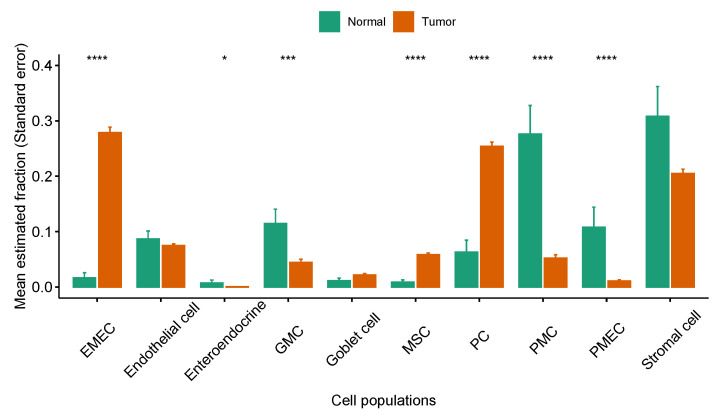
Comparison of the relative abundance of non-immune cell populations between normal and tumor samples in the TCGA-STAD cohort, * p<0.05; *** p<0.001; **** p<0.0001.

We hypothesized that the EMEC population would be more abundant in patients with stage I cancer than other stages. To test this hypothesis, we estimated proportions of the EMEC population in each sample of both the ACRG and TCGA-STAD cohorts. We observed significantly higher EMEC populations in patients with stage I cancer than stage II, III, or IV in both the ACRG and TCGA-STAD cohorts (p<0.05, Student’s *t*-test; Figure 7). To investigate whether the EMEC population was typically found in tumor samples but not in normal samples, we estimated proportions of 10 non-immune cell populations in samples from both tumor and adjacent normal tissues available in the TCGA-STAD sample collection. We observed that the EMEC population was significantly higher in tumor samples than in adjacent normal samples (p<0.0001, Student’s *t*-test; Figure 8).

We also observed the PMEC population was significantly lower in tumor samples than in normal samples (p<0.0001, Student’s *t*-test; Figure 8). The proliferative cell type was the second major epithelial cell type found in tumors. It was significantly increased in tumor samples when compared to normal samples (p<0.0001, Student’s *t*-test; Figure 8). By contrast, the PMC cell types were significantly decreased in tumor samples compared to normal samples (p<0.0001, Student’s *t*-test; Figure 8).

### 3.3. Correlates of Non-Immune/Immune Factors with Overall Survival

**Figure 9:**
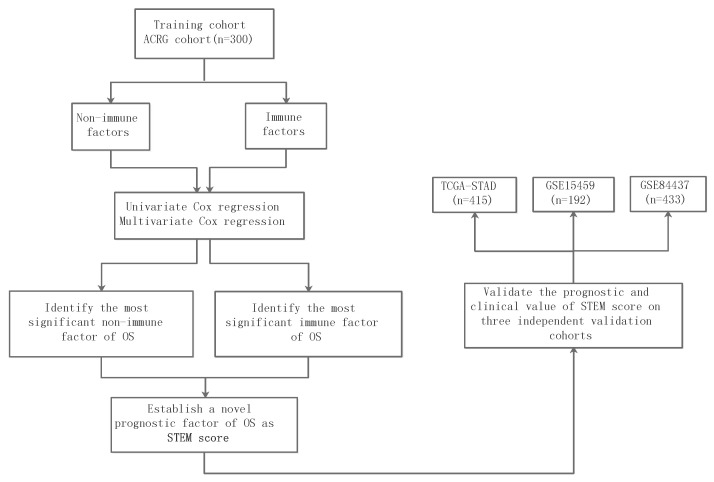
Schema of study design.

We developed a comprehensive and systematic analysis of diverse epithelial, stromal, and immune cell types within the TME and their associations with GC risk (Figure 9). The ACRG (GSE62254) cohort was used as the training cohort because it provided the most comprehensive clinical data along with more than five years of follow-up information for 300 GC patients. The univariate Cox proportional hazards regression model was used to identify prognostic factors of overall survival (OS) in the training cohort. We further expanded the univariate analyses to multivariate Cox proportional hazard analyses, which accounts for age, sex, stage, Lauren histology, and adjuvant chemotherapy treatment as additional clinical covariates to examine their independent prognostic values. We performed univariate Cox analyses to evaluate all non-immune (or immune) factors that were defined by calculating the ratios of relative fractions in each pair of non-rare non-immune (or immune) cell populations that consisted of larger than 5% of the total non-immune (or immune) cell population. All non-immune or immune factors with a hazard ratio (HR) ≠1 identified using univariate Cox regression analyses are shown in Table 1 and Table 2, respectively. The univariate Cox analyses indicated that the three non-immune factors of EMEC/stromal cell, PC/stromal cell, and endothelial cell/stromal cell ratios were significantly correlated to OS, and the hazard ratio ranged from 0.27 to 0.8 (p<0.05). Additionally, the ratios of EMEC/stromal cell and PC/stromal cell remained significant prognostic factors of OS by multivariate analyses, with hazard ratios of 0.82 and 0.85 (p<0.05), respectively (Table 1). The univariate Cox regression analysis for the prediction of OS also confirmed that three immune factors—the ratios of T adaptive/monocytes, monocytes/T adaptive, and monocytes/B adaptive were all found to be of prognostic significant factors with a hazard ratio ranging from 0.86 to 2.54 (p<0.05). The multivariate analyses confirmed that the T adaptive/monocytes and monocytes/T adaptive ratios were independent prognostic factors of OS with hazard ratios of 0.88 and 2.61 (p<0.05), respectively (Table 2).

Thus, we defined a new TME score, called the STEM score for each GC sample by combining the most significant non-immune (the ratio of EMECs to stromal cells) and immune factors (the ratio of adaptive T cells to monocytes) as:

(1) $$\mathrm{STEM}\;\mathrm{score}=\frac{\mathrm{EMEC}}{\mathrm{Stromal}\;\mathrm{cell}}+\frac{T\;\mathrm{adaptive}}{\mathrm{Monocytes}}$$

We performed multivariate Cox regression analysis of the STEM score correcting for clinicopathological variables, including age, sex, stage, Lauren histology, and adjuvant chemotherapy treatment. We found that the STEM score acts as an independent prognostic factor for OS (HR 0.9, p=0.003) in the training cohort (Table 3).

**Figure 10:**
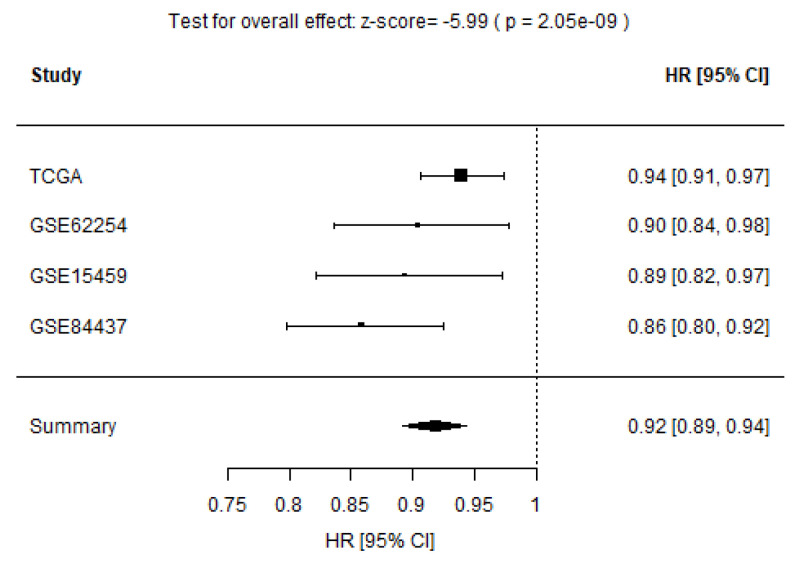
Forest plot of estimated hazard ratios indicating STEM score as a prognostic factor for OS in four GC cohorts.

To validate whether the STEM score had consistent prognostic value in different cohorts, we applied it to three independent validation data sets from TCGA-STAD, GSE15459, and GSE84437. The multivariate Cox proportional hazard analyses, which account for age, sex, stage, Lauren histology, and adjuvant chemotherapy/radiation therapy treatment (if applicable as additional clinical covariates) confirmed that the STEM score was an independent prognostic factor of OS in each validation data set (TCGA-STAD: HR 0.94, p=0.001, GSE15459: HR 0.89, p=0.01, and GSE84437: HR 0.86, p<0.001; Table 4). We further performed a meta-analysis to evaluate the overall effect of the STEM score on clinicopathologic factor-adjusted survival. We added three validation cohorts to the training cohort. A forest plot of estimated hazard ratios indicated the STEM score was a significant independent prognostic factor of OS (HR = 0.92, $p=2.05\times 10^{-9}$, z-test for overall effect; Figure 10).

### 3.4. Increased Stem Score Associated with Superior Survival

**Figure 11:**
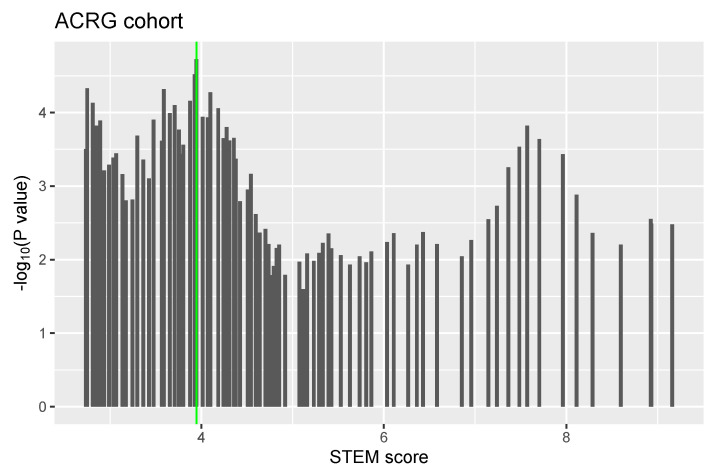
An illustration of optimal cutoff identification for STEM score.

**Figure 12:**
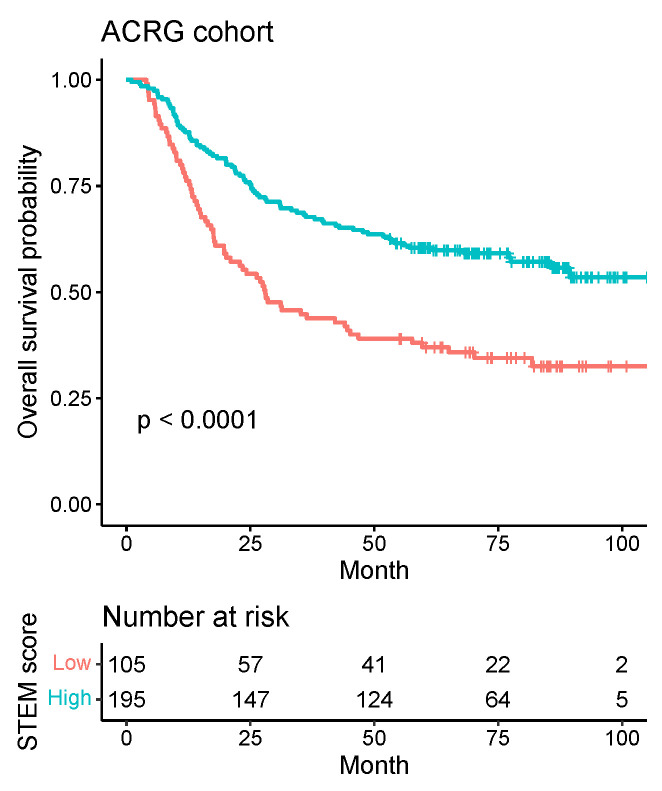
Kaplan–Meier survival curves for the patients with low vs. high STEM scores in the ACRG cohort.

We next assessed the predictive value of the STEM score for risk stratification. For the ACRG cohort, we stratified 300 patients into two groups (low vs. high) according to the STEM score and using an optimal cutoff of 3.95 (Figure 11; see Section 2 for details). The Kaplan–Meier curve showed that patients in the high-STEM score group had significantly longer OS times than patients in the low-STEM score group (p<0.0001, log-rank test; Figure 12).

**Figure 13:**
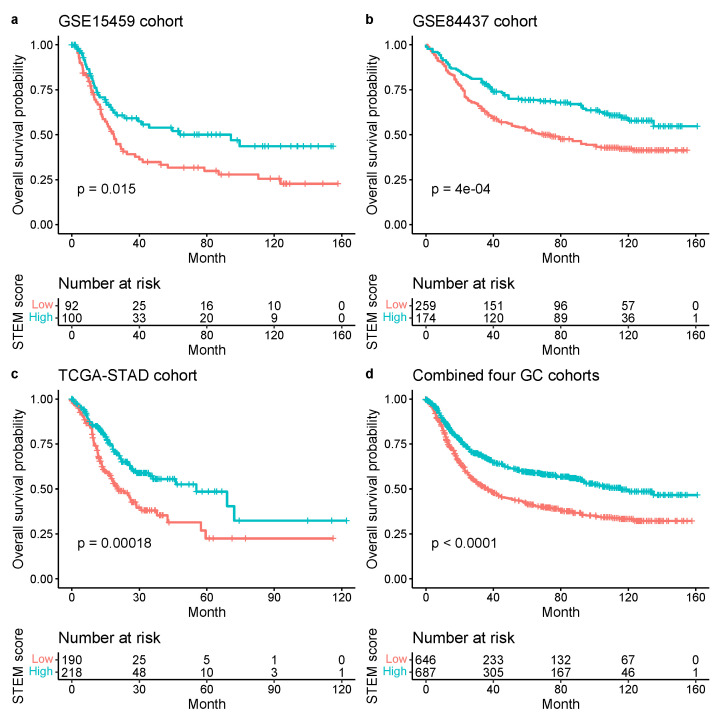
Kaplan–Meier plots for the OS of patients with low vs. high STEM scores in the GSE15459 (**a**), GSE84437 (**b**), TCGA-STAD (**c**) cohorts and a combined set of four cohorts (ACRG, GSE15459, GSE84437, and TCGA-STAD) (**d**). Significance test *p* value is shown in the lower left.

By using the same cutoff optimized in the ACRG cohort, we observed consistent results across three independent validation cohorts that showed patients with a high STEM score yielded better OS than those with a low STEM score (GSE15459: p=0.015; GSE84437: $p=4\times 10^{-4}$; TCGA-STAD: $p=1.8\times 10^{-4}$; a combined set of four cohorts: p<0.0001; log-rank test, Figure 13).

**Figure 14:**
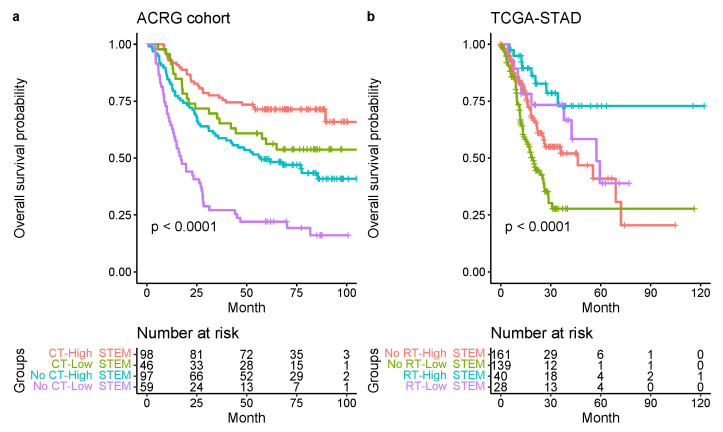
(**a**) Kaplan–Meier survival curves for the four groups (including chemotherapy-high STEM score, chemotherapy-low STEM score, no chemotherapy-high STEM score, and no chemotherapy-low STEM score) in the ACRG cohort. Significance test *p* value is shown in the lower left. (**b**) Kaplan–Meier survival curves for the four groups (including radiation therapy-high STEM score, radiation therapy-low STEM score, no radiation therapy-high STEM score, and no radiation therapy-low STEM score) in the TCGA-STAD cohort.

Additionally, we investigated the prognostic values of the STEM score in groups of patients treated with or without chemotherapy. We then stratified the ACRG cohort into four groups based on the STEM score and chemotherapy (CT) treatment or not. The unadjusted survival curve for the four groups indicated the high-STEM score groups had superior survival compared to the low-STEM score group for patients regardless of chemotherapy treatment (Figure 14a).

To explore the prognostic values of the STEM score in groups of patients treated with or without radiation therapy, we stratified the TCGA-STAD cohort into four groups based on the STEM score and radiation therapy (RT) treatment or not. The unadjusted survival curve for the four groups indicated the high-STEM-score groups still had superior survival compared to the low-STEM-score group for patients regardless of radiation therapy treatment (Figure 14b).

**Figure 15:**
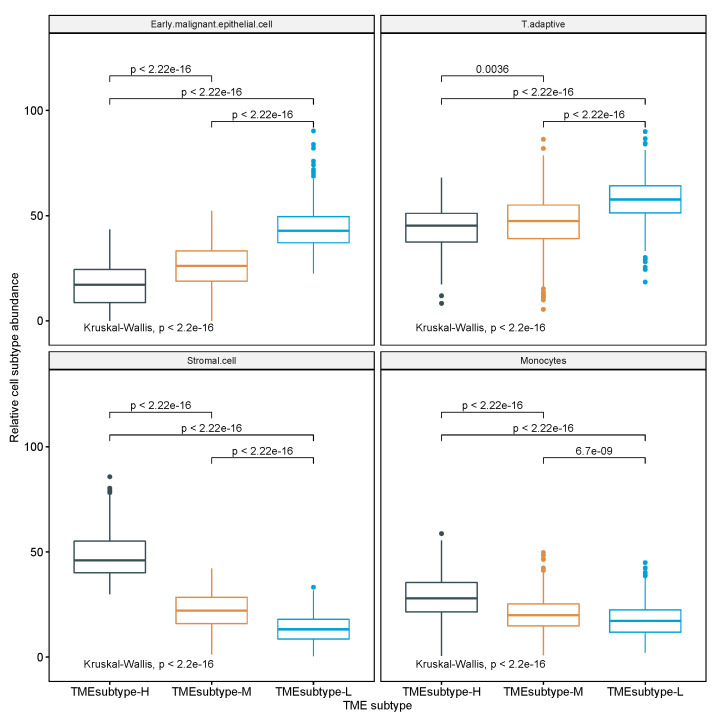
Comparison of the estimated proportions of four prognostic cell populations across three TME subtypes in the meta cohort.

By using multivariate Cox regression analysis and univariate Kaplan–Meier analysis in Section 3.3, four prognostic cell populations of Stromal cell, adaptive T cell, EMEC, and monocytes, referred to as STEM populations, that had been found to be significant in the univariate Cox regression analyses were further examined to be prognosis stratification factors in gastric cancer. Thus, these four cell populations were selected to perform cluster analysis of all 1340 GC samples in four GC cohorts. Based on the estimated relative abundance of these four cell populations, we applied spectral clustering with the optimal number of clusters chosen by NbClust (see Section 2 for details). The three resulting TME subtypes, including TMEsubtype-H, TMEsubtype-M, and TMEsubtype-L (with 302, 591, and 447 samples, respectively) were characterized by a distinct distribution of relative abundance over the four selected cell populations. The relative abundance of these four cell populations varied significantly across the three TME subtypes ($p<2.22\times 10^{-16}$, Kruskal–Wallis test; Figure 15). The TMEsubtype-H cluster had the highest stromal cell (mean proportion = 0.48, $p<2.22\times 10^{-16}$, Wilcoxon test relative to the next-highest; Figure 15, left bottom) and monocyte abundance (mean proportion = 0.29, $p<2.22\times 10^{-16}$, Wilcoxon test relative to the next-highest; Figure 15, right bottom), while it had the lowest EMEC (mean proportion = 0.17, $p<2.22\times 10^{-16}$, Wilcoxon test relative to the next-lowest; Figure 15, left top) and adaptive T cell abundance (mean proportion = 0.44, p=0.0036, Wilcoxon test relative to the next-lowest; Figure 15, right top). However, in the TMEsubtype-L, the opposite was observed. The TMEsubtype-L cluster was found to have the lowest stromal cell (mean proportion = 0.14, $p<2.22\times 10^{-16}$, Wilcoxon test relative to the next-lowest; Figure 15, left bottom) and monocyte abundance (mean proportion = 0.18, $p=6.7\times 10^{-9}$, Wilcoxon test relative to the next-lowest; Figure 15, right bottom), while it had the highest EMEC (mean proportion = 0.44, $p<2.22\times 10^{-16}$, Wilcoxon test relative to the next-highest; Figure 15, left top) and adaptive T cell abundance (mean proportion = 0.57, p=0.0036, Wilcoxon test relative to the next-highest; Figure 15, right top). The mean proportions of EMEC, stromal cell, adaptive T cell, and monocytes for the TMEsubtype-M were 0.25, 0.22, 0.47, and 0.2, respectively.

**Figure 16:**
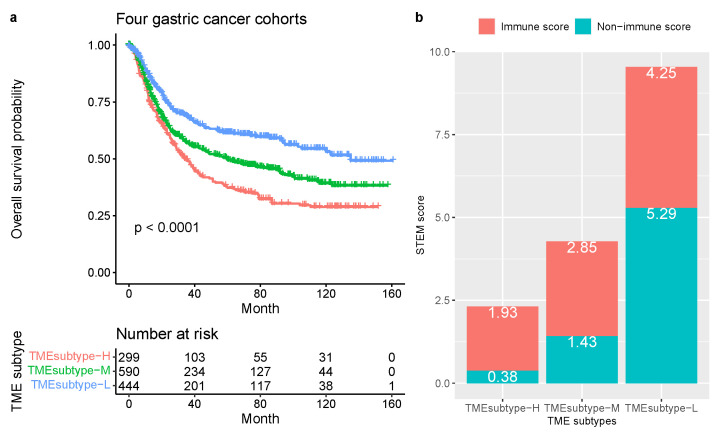
TME subtypes in gastric cancer. (**a**) Kaplan–Meier plots for the OS of patients stratified into three TME subtypes in the meta cohort (ACRG, GSE15459, GSE84437, and TCGA-STAD). Significance test *p* value is shown in the lower left. (**b**) The mean non-immune and immune scores with TME subtypes.

**Figure 17:**
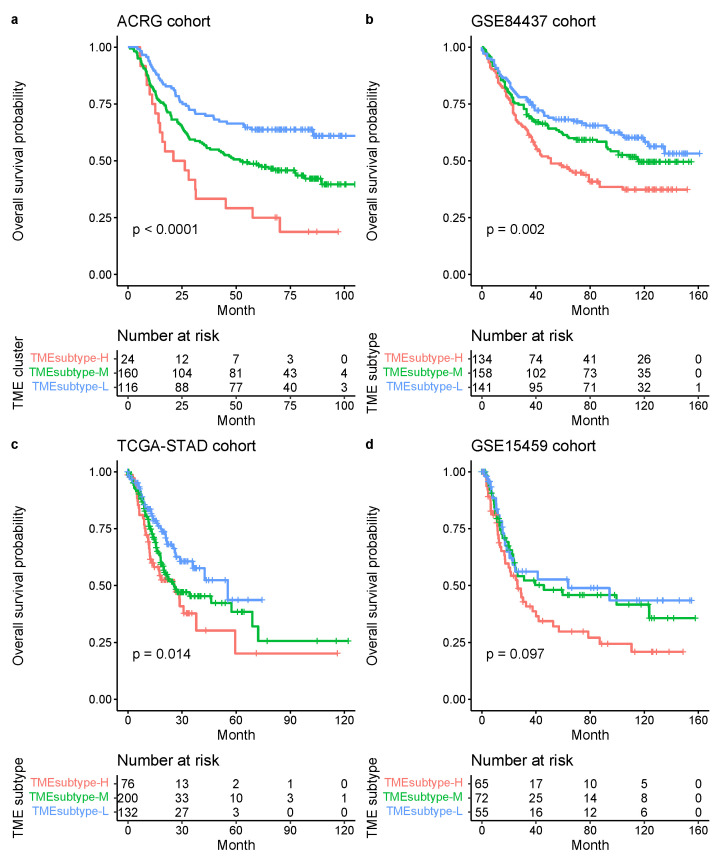
Kaplan–Meier plots for the OS of patients stratified into three TME subtypes in the ACRG (**a**), GSE84437 (**b**), TCGA-STAD (**c**), and GSE15459 (**d**) cohorts. Significance test *p* value is shown in the lower left.

We further examined the TME subtypes’ association with OS. The TMEsubtype-L had the best prognosis (OS HR (95% CI) 0.52 (0.42–0.65), p<0.0001 relative to the TMEsubtype-H, adjusted for age and sex); the TMEsubtype-M had intermediate prognosis (OS HR (95% CI) 0.76 (0.63–0.91), p=0.0035 relative to the TMEsubtype-H, adjusted for age and sex); and the TMEsubtype-H had the least favorable outcome (p<0.0001, log-rank test; Figure 16a). A decreased value of the STEM, non-immune, or immune score led to worse outcomes in the TMEsubtype-H (Figure 16b). The survival analysis revealed a substantial difference in OS among the three TME subtypes. Robust correlations between the identified TME subtypes and OS were also validated in ACRG (p<0.0001, log-rank test; Figure 17a), GSE84437 (p=0.002, log-rank test; Figure 17b), and TCGA-STAD (p=0.014, log-rank test; Figure 17c) cohorts, separately. Patients in different GC cohorts were stratified into three groups with significantly distinct prognoses, and it was found that the lower the STEM score the poorer the patient survival outcome. Although the difference in the GSE15459 cohort was not statistically significant (p=0.097, log-rank test; Figure 17d), the TMEsubtyp-H still had the poorest outcome.

**Figure 18:**
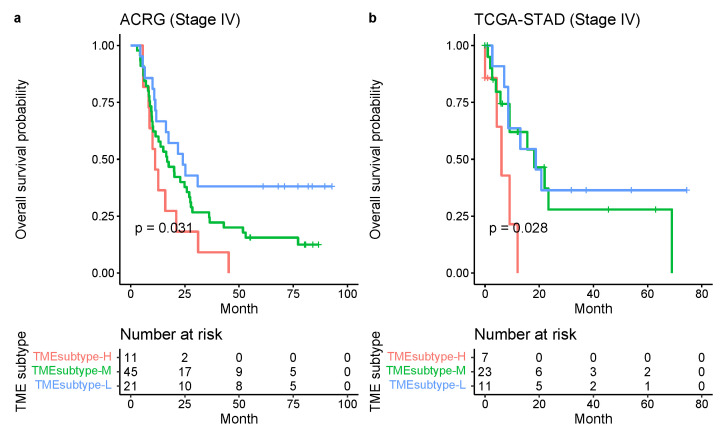
TME subtype in patients with stage IV GC. Kaplan–Meier plots for the OS of patients with stage IV GC stratified into three TME subtypes in the ACRG (**a**) and TCGA-STAD (**b**) cohorts.

The risk stratification model was further investigated in patients with the same stage GC in the ACRG and TCGA-STAD data sets. Similarly, to the whole cohort, patients with stage IV GC were stratified into three groups with significantly distinct prognoses (ACRG: p=0.031, Figure 18a and TCGA-STAD: p=0.028, Figure 18b; log-rank test).

**Figure 19:**
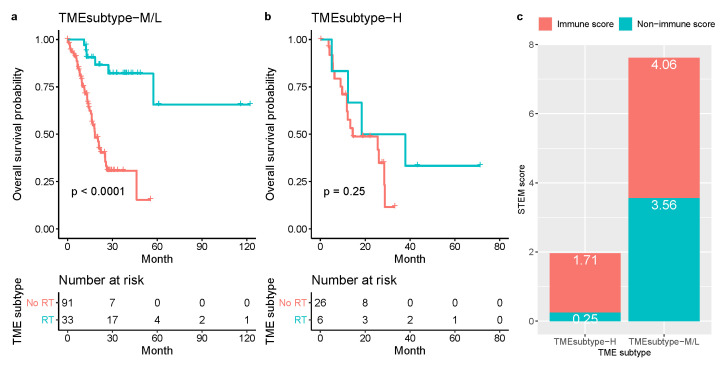
Relationship between the TME subtypes and survival benefit from radiation therapy in matched patients with stage III gastric cancer: (**a**) TMEsubtype-H, (**b**) TMEsubtype-M/L. (**c**) Compare the mean non-immune and immune scores of patients in the TMEsubtype-H to the TMEsubtype-M and -L for stage III disease in the TCGA-STAD cohort.

To investigate the predictive value of the TME subtypes for radiation therapy response in the TCGA-STAD cohort, we evaluated the association between TME subtypes and overall survival among stage III patients who either received or did not receive radiation therapy. We found that for patients in the TMEsubtype-M and TMEsubtype-L group, radiation therapy was associated with improved OS (HR 0.16, 95% CI (0.06–0.4), p<0.0001; Figure 19a). However, for patients in the TMEsubtype-H group, performing radiation therapy did not improve OS (HR 0.49, 95% CI (0.14–1.72), p=0.25; Figure 19b). The mean STEM score of patients in the TMEsubtype-H group was substantially smaller than those in the TMEsubtype-M and TMEsubtype-L groups (Figure 19c).

### 3.5. Comparison with Other Reported Molecular Classifications for GC

**Figure 20:**
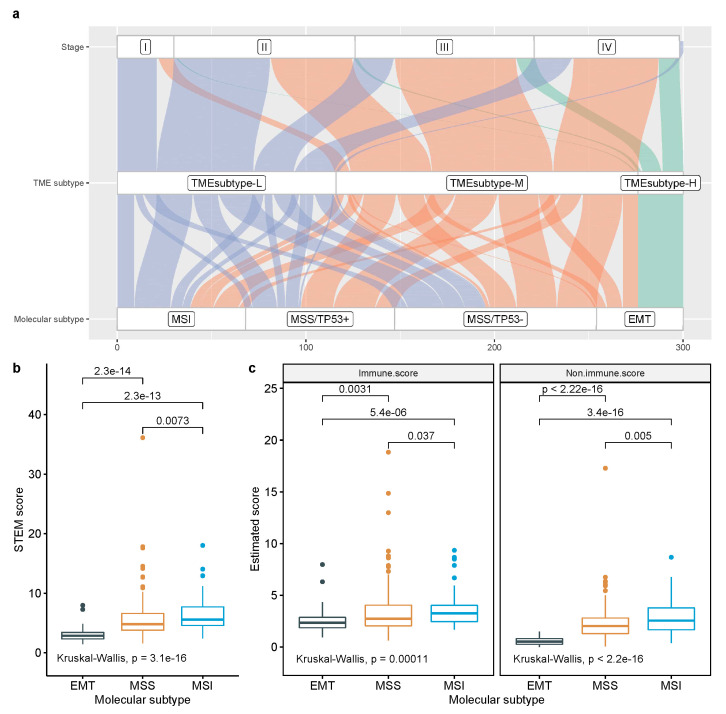
Association of TME subtypes and ACRG molecular subtypes. (**a**) Alluvial plot displaying the association of TME subtypes, ACRG molecular subtype, and stage. (**b**) Comparison of STEM score across three ACRG molecular subtypes. (**c**) Comparison of non-immune (e.g., ratio of EMEC/stromal cell) and immune scores (e.g., ratio of T adaptive/monocytes) across three ACRG molecular subtypes.

We compared the similarities and differences of the identified TME subtypes with the molecular subtypes derived by the ACRG, as well as with the genomic subtypes derived by the TCGA. The ACRG classified GC into four molecular subtypes, including EMT, MSS/TP53-, MSS/TP53+, and MSI, which are associated with distinct molecular alterations, disease progression, and survival outcomes based on gene-expression data. In survival analysis, the MSI subtype had the best prognosis, followed by MSS/TP53+, MSS/TP53−, and the EMT subtype having the worst prognosis [4]. The comparisons of the TME subtypes with the ACRG molecular subtypes showed several differences; for instance, the samples classified as the ACRG EMT subtype were present in both the TMEsubtype-H and TMEsubtype-M, and the samples classified as ACRG MSS (TP53+ and TP53−) and MSI subtypes were present in both the TMEsubtype-M and TMEsubtype-L. However, we observed that the TMEsubtype-H, TMEsubtype-M, and TMEsubtype-L were enriched in ACRG EMT, MSS (TP53+ and TP53−), and MSI, respectively (Figure 20a). Moreover, we investigated the association of the TME subtypes with tumor stages. The TMEsubtype-H was linked to patients classified as stage III and IV, whereas the TMEsubype-L was associated with patients classified as early stage I and II (Figure 20a). We showed previously that a lower STEM score was associated with a poorer survival outcome. We found similar results when comparing the STEM score across ACRG molecular subtypes. The ACRG EMT subtype, composed mostly of diffuse-type tumors, had been shown to have the worse prognosis of the four and was linked to the lowest STEM score (mean STEM score = 3.1, $p=2.3\times 10^{-14}$, Wilcoxon test relative to next-lowest), indicating the ACRG EMT subtype was enriched with patients predicted to have higher stromal and monocyte abundance and lower EMEC and adaptive T cell abundance. The ACRG MSI subtype, which had been shown to have the best prognosis of four, was linked to the highest STEM score (mean STEM score = 6.49, p=0.0073, Wilcoxon test relative to next-highest), indicating the ACRG MSI subtype was enriched with patients predicted to have lower stromal and monocyte abundance and higher EMEC and adaptive T cell abundance. The mean STEM score of the ACRG MSS subtype was 5.73. The STEM score varied significantly across the four ACRG molecular subtypes ($p=3.1\times 10^{-16}$, Kruskal–Wallis test; Figure 20b). Consistent results were found for the non-immune and immune scores across the ACRG molecular subtypes (Figure 20c). Further, to ask whether the identified TME subtypes provide complementary prognostic value to the ACRG molecular subtype, we investigated the risk stratification model in patients with the same ACRG molecular subtype (Figure S5). Patients within the ACRG MSS/TP53- subtype were stratified into two groups with significantly distinct prognoses (p=0.013, Figure S5c).

**Figure 21:**
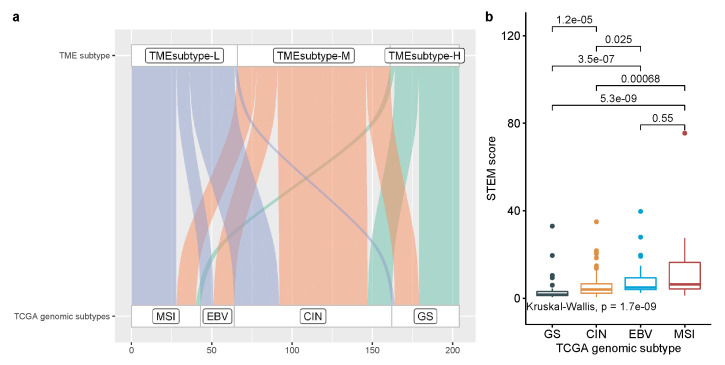
Association of TME subtypes and TCGA genomic subtypes. (**a**) Alluvial plot displaying the association of TME subtypes and TCGA-STAD subtypes. (**b**) Comparison of STEM score across four TCGA genomic subtypes.

The TCGA research network classified GC into four genomic subtypes, including EBV, MSI, GS, and CIN, by integrating data from six molecular platforms and performing Microsatellite instability (MSI) testing [5]. The MSI and EBV subtypes were shown to have a better prognosis than GS and CIN subtypes [4,34,35]. The comparisons of the TME subtypes with the TCGA genomic subtypes showed similarities, such as the TMEsubtype-H, TMEsubtype-M, and TMEsubtype-L being enriched in TCGA GS, CIN, and MSI, respectively (Figure 21a). The TMEsubtype-H is primarily composed of samples classified as the TCGA GS and CIN subtypes. The GC samples classified as TMEsubtype-M were present across all TCGA genomic subtypes. The TMEsubtype-L is primarily composed of samples classified as the TCGA MSI, EBV, and CIN subtypes. We found a significantly lower mean STEM score in the TCGA GS (3.57) and CIN (5.7) subtypes compared to the EBV (9.41) and MSI (11.4) subtypes (Figure 21b), further reinforcing the prognostic value of the STEM score. Importantly, we found that the TME subtypes also provided complementary prognostic value to the TCGA genomic subtypes (Figure S6). In the patients with undefined TCGA subtype, they were stratified into two groups with significantly distinct prognoses (p=0.00061, Figure S6b). Within the TCGA CIN subtype, which is known to have unfavorable outcomes, our TME subtypes can still distinguish subgroups of patients with distinct prognoses (p=0.011, Figure S6d).

### 3.6. Identification of GC Prognostic Gene Signatures

We showed predictive values of the EMEC and stromal cell populations for OS. In this respect, we further investigated prognostic gene signatures of these two cell populations. By using scRNA-seq transcription profiles, we identified 150 gene markers for the EMEC and stromal cell populations on the construction of the non-immune signature matrix. For each marker gene, we did multivariate Cox proportional hazard modeling on the four GC cohorts, respectively, accounting for conventional clinical and pathologic factors including age, sex, stage, Lauren histology, and adjuvant chemotherapy/radiation therapy treatment if applicable. Next, we performed fixed-effects meta-analyses to identify gene signatures whose expressions were significantly associated with survival outcome across multiple cohorts.

**Figure 22:**
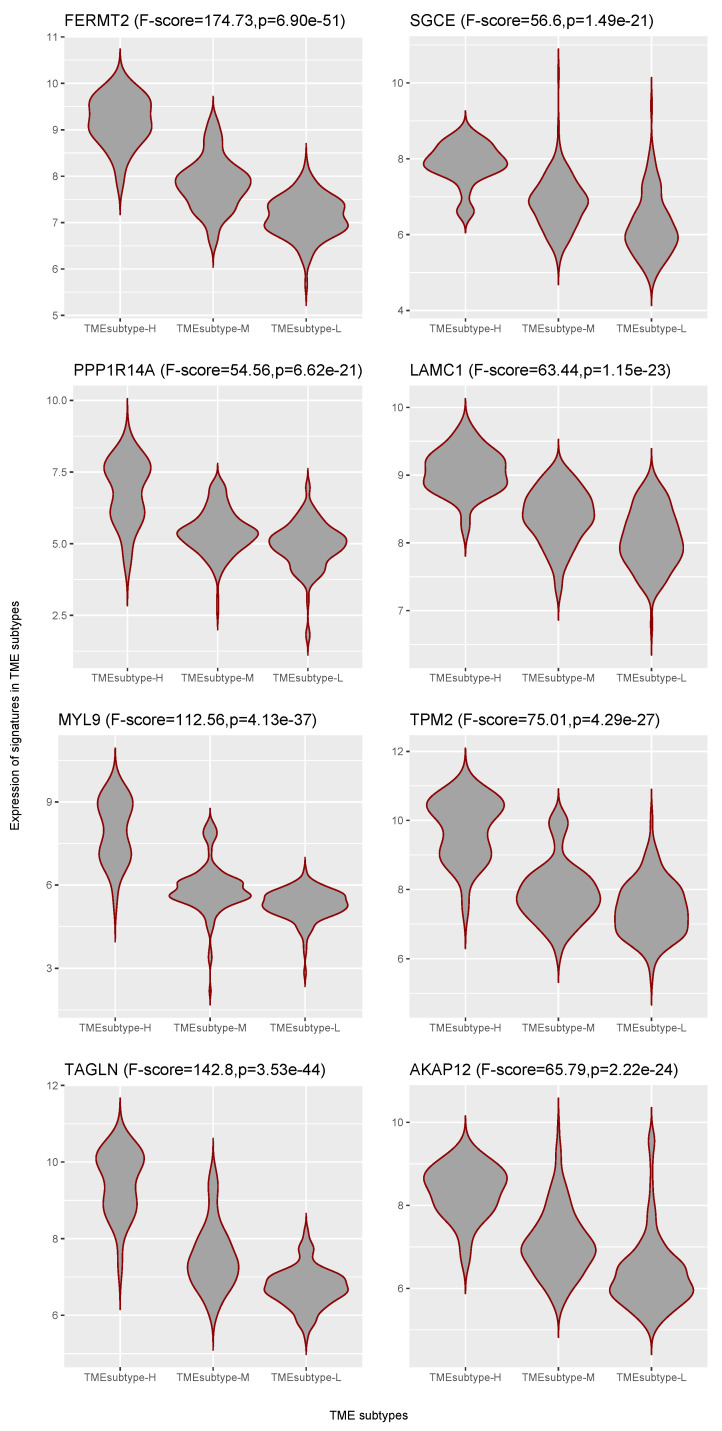
Violin plots showing differential expression of representative stromal cell markers in three TME subtypes.

A higher abundance of the stromal cell subtype has been associated with poorer prognosis. Thus, we identified those prognostic genes for each GC cohort at a *p*-value cutoff of 0.05, as well as those with a hazard ratio greater than 1, which suggested a significant increase in risk. There were 43 significant prognostic gene signatures detected after performing the meta-analysis ($p<10^{-5}$, z-test for overall effect). The top eight genes, including FERMT2 (Fermitin family homolog 2, HR = 1.49), SGCE (sarcoglycan epsilon, HR = 1.5), PPP1R14A (protein phosphatase 1 regulatory inhibitor subunit 14A, HR = 1.38), LAMC1 (laminin subunit gamma 1, HR = 1.83), MYL9 (myosin light chain 9, HR = 1.3), TPM2 (tropomyosin 2, HR = 1.34), TAGLN (transgelin, HR = 1.33), and AKAP12 (A-kinase anchoring protein 12, HR = 1.4), that had a smaller *p* value for the overall effect are shown in Table 5 and Figure S7. We further explored the association between the expression levels in prognostic marker genes with TME subtypes. We focused on the ACRG cohort. Violin plots indicate that the representative prognostic marker genes were highly expressed in TMEsubtype-H with relatively low levels in the other two TME subtypes: TMEsubtype-M and TMEsubtype-L (Figure 22). The expression levels were significantly different among three TME subtypes ($p<10^{-21}$, one-way ANOVA F test).

**Figure 23:**
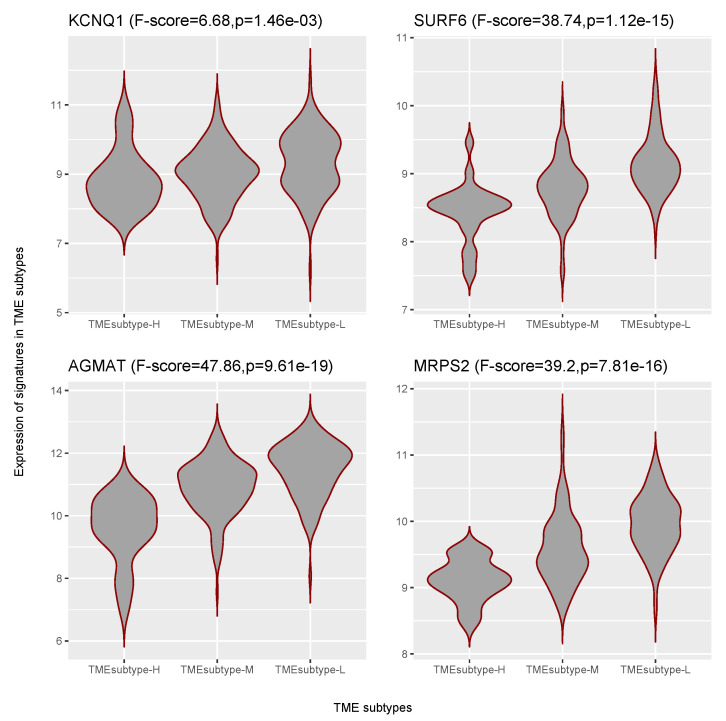
Violin plots showing differential expression of representative EMEC markers in three TME subtypes.

The EMEC subtype showed an opposite trend (Table 5). A lower abundance of that subtype has been shown to be associated with poorer prognosis. We therefore identified the prognostic genes for each GC cohort at a *p*-value cutoff of 0.05, as well as with a hazard ratio below 1, which suggests a significantly smaller risk. No significant prognostic genes were found across all four GC cohorts; therefore, we just kept the genes that were significantly prognostic across three of the four GC cohorts for further analysis. Finally, four significant prognostic gene signatures, including KCNQ1 (potassium voltage-gated channel subfamily Q member 1, HR = 0.73), SURF6 (surfeit 6, HR = 0.57), AGMAT (agmatinase, HR = 0.79), and MRPS2 (mitochondrial ribosomal protein S2, HR = 0.6), were detected after performing meta-analysis ($p<10^{-5}$, z-test for overall effect; Figure S8). Violin plots for the ACRG cohort indicated the representative prognostic marker genes were highly expressed in TMEsubtype-L with relatively low levels in the other two TME subtypes (Figure 23). The expression levels were significantly different among three TME subtypes (p<0.001, one-way ANOVA F test).

## 4. Discussion

In the present study, a comprehensive and systematic analysis of diverse epithelial, stromal, and immune cell types within the TME and their associations with GC risk was developed. We examined several large cohorts of GC patients at the cellular level and found a new and strong independent prognostic factor (STEM score) for GC patients. The STEM score was defined as the arithmetic sum of the two most significant TME factors: the EMEC-to-stromal ratio and the adaptive-T-cell-to-monocyte ratio. Our results suggest that high-risk patient groups (STEM score ≤3.95) had significantly shorter OS times than patients in the low-risk group (STEM score >3.95). The STEM score includes four major epithelial, stromal, and immune cell populations and greatly facilitated the quantitative characterization of GC TME in a comprehensive manner.

Stromal cells, especially cancer-associated fibroblasts (CAFs), in the TME have been found to promote growth and survival of malignant cells [36]. Many studies have found that cancer cells release factors promoting fibroblasts to secrete tumor-promoting chemokines [37]. The interactions of tumors and CAFs can lead to increased malignancy in many cancer types [38,39]. Several studies of GC suggest that a low tumor-to-stroma ratio (TSR) is associated with a poor prognosis [19,40]. Herein, we analyzed a gastric scRNA-seq data set that covered diverse epithelial cell types isolated from patients with NAG, CAG, IM, and EGC, and identified the EMEC population. The EMEC-to-stromal cell ratio was shown to have significant correlation to OS, which agreed with previous studies [19,40] on the positive prognostic value of TSR. The EMEC population is comprised of cancer cells, MSCs, PCs, and PMCs that emerged uniformly in the EGC biopsy and that were predominantly present in tumor samples but not typically found in adjacent normal samples. Additionally, significantly higher EMEC populations were detected in patients with stage I cancer than stage II, III, or IV, suggesting the value of the EMEC population in the early detection of gastric cancer.

There is increasing evidence that suggests a strong infiltration of T cells, especially CD8+ T cells, into the TME correlates with a good prognosis in many types of cancer, and this has implications for the success of active cancer immunotherapy [41,42]. Studies have shown that CD8+ T cells play a vital role in mediating anti-tumor immunity, and cytotoxic CD8+ memory T cells kill tumor cells by recognizing tumor-associated antigens presented on major histocompatibility complex class I [43,44,45]. High numbers of CD4+ T helper 1 cells in the TME also correlate with a better prognosis [45]. Tumor-associated macrophages were found to enhance malignant cell migration, invasion, and metastases [46]. Monocytes can give rise to macrophages, so the abundance of monocytes may lead to increased production of macrophages. In our study of GC cohorts, an increased adaptive T cell to monocyte ratio was significantly associated with increased OS. This is in line with studies by [47] of haematologic malignancies and [48] of stage III colon cancer. The studies of haematologic malignancies and stage III colon cancer demonstrate that an elevated lymphocyte-to-monocyte ratio (LMR) yields a better survival outcome.

Molecular signatures associated with distinct clinical outcomes have been studied in many types of cancer [5,49,50]. We identified several gene signatures of the EMEC and stromal cell populations to be independent prognostic factors of OS in multivariate analysis. Several prognostic gene signatures of stromal cells have been previously reported to play oncogenic roles in cancer cell proliferation, migration, or invasion. FERMT2 (also known as Kindlin-2), a focal adhesion protein, has been found to regulate cancer cell proliferation, apoptosis, and chromosomal abnormalities in breast cancer that are associated with tumor stromal invasion, lymph node metastasis, and patient outcome in gastric cancer. Over-expression of FERMT2 promotes tumor formation in breast cancer and was linked with poorer patient outcomes [51,52]. TAGLN is expressed in fibroblasts and smooth muscle, and the overexpression of TAGLN has been found in the tumor-induced reactive myofibroblastic stromal tissue in lung adenocarcinoma, as well in carcinomas of the stomach, liver, and oesophagus [53]. Silencing of TAGLN2, a homologue of TAGLN, has been reported to significantly inhibit cell proliferation and increase apoptosis in bladder cancer [54]. MYL9 was previously found to be over-expressed in stages III and IV of non-small-cell lung cancer [55]. Over-expression of MYL9 in tumor cells was associated with poorer OS and recurrence-free survival in esophageal squamous cell carcinoma [56]. TPM2, a marker of fibroblasts, was previously reported to be associated with poor prognosis in colorectal cancer (CRC) [57]. The TAGLN, MYL9, and TPM2 were found to be over-expressed in fibroblasts from primary tumors compared to adjacent normal tissues and were associated with a poorer prognosis in the TCGA cohort of colorectal cancer [57]. PPP1R14A, also known as CPI-17, has been investigated as a prognostic biomarker of gastric cancer [58]. Previous studies have revealed that PPP1R14A can drive Ras activity and promote tumorigenic transformation [59,60]. LAMC1 was found to be a target of miR-29s. Silencing of LAMC1 significantly inhibited cell migration and invasion in prostate cancer cells [61]. AKAP12 has been investigated as a tumor suppressant in some human primary cancers, including GC [62,63]; however, in the present study, we found it significantly over-expressed in the TMEsubtype-H high-risk group, suggesting an increased risk of OS with higher expression levels.

Herein, we identified four prognostic gene signatures of the EMEC population to be positively associated with OS. AGMAT were found to be positively associated with OS in kidney renal clear cell carcinoma [64]. MRPS2 encoding the mitochondrial ribosomal protein S2 was important for mitoribosome formation and stability and mitochondrial translation. It was reported to predict poor OS in ovarian cancer patients [65]. However, we found that it was associated with better clinical outcomes in GC patients. KCNQ1 has been shown to distribute widely and be functionally relevant in a variety of epithelial tissues [66]. There is preliminary evidence to suggest that KCNQ1 is a tumor suppressor in the stomach and colon [67,68]. Low or loss of expression of KCNQ1 was previously found to associate with poor disease-free survival in stage II and III colon cancer patients [69]. Moreover, over-expression of KCNQ1 in the colorectal cancer cell line was found to have trapped β-catenin at the plasma membrane, induced a patent lumen in CRC spheroids, and slowed CRC cell invasion [68].

In addition, many studies have revealed some gene signatures with potential therapeutic value in cancers. LAMC1 has recently been found to be overexpressed in endometrial cancer and is reported to be a potent biomarker for identifying endometrial cancer patients needing aggressive adjuvant therapy [70]. Several studies have demonstrated that TAGLN2 expression in cancer cells is associated with increased drug resistance, and selectively suppressed TAGLN2 expression may prevent multidrug resistance in cancer chemotherapy [71,72,73,74]. Over-expression of FERMT2 was previously found to promote melanoma growth and migration, which was attributed to stimulate the downstream MAPK pathway, and was reported to be a potential therapeutic target for treating melanoma [75].

Strengths of our study include the use of multiple independent validation cohorts, and the use of three transcriptome profiling technologies, including microarray, bulk RNA-seq, and scRNA-seq, which improve the reliability and generalizability of the results. We created and validated the GC10 signature matrix for deconvolution of 10 epithelial and stromal subsets in gastric samples, which enabled the investigation of the heterogeneous tumor microenvironment in GC. Nevertheless, our study had several limitations. The main limitation of this study was the retrospective nature, which might have been subject to selection bias. Moreover, we characterized the cell populations using only gene expression measurements; high-resolution mass-spectrometry-based proteomics profiling may reduce the potential bias of transcriptome profiling technologies. The retrospective nature of this study calls for further validation using a prospective investigation.

In conclusion, we identified significant prognostic factors and gene signatures among diverse epithelial, stromal, and immune cell populations in the GC TME. Our findings indicated that assessment of the early malignant epithelial, stromal, adaptive T cells, and monocyte abundance via the STEM score provided a potent predictor of survival in patients with GC. This study demonstrated the STEM score as an independent prognostic factor for GC. It showed that a lower STEM score was significantly associated with a shorter survival time. The entire GC cohort was stratified into three risk groups (high-, moderate-, and low-risk) based on the four STEM populations, which yielded incremental survival times. The risk stratification model may aid stratification of patients with stage III gastric cancer for radiation therapy and may be informative for refinement of molecular subtypes of GC.

## Figures and Tables

**Table 1:** Univariate and multivariate results in Cox proportional hazards analysis of non-immune factors in the training cohort. The *p* value was derived from the Cox regression model; FDR adjusted, multiple comparison adjusted *p* values calculated by false discovery rate approach; HR, hazard ratio; CI, confidence interval.

	Univariate Analysis		Multivariate Analysis	
**Non-Immune Factors**	**HR (95% CI for HR)**	***p*** **Value (FDR-Adjusted)**	**HR (95% CI for HR)**	***p*** **Value (FDR-Adjusted)**
EMEC/Stromal cell	0.75 (0.65–0.86)	<0.001 (<0.001)	0.82 (0.71–0.94)	0.005 (0.022)
PC/Stromal cell	0.80 (0.71–0.90)	<0.001 (<0.001)	0.85 (0.75–0.96)	0.011 (0.022)
Endothelial cell/Stromal cell	0.27 (0.08–0.90)	0.032 (0.064)	0.44 (0.13–1.47)	0.182 (0.273)
Stromal cell/Endothelial cell	1.04 (1.00–1.08)	0.081 (0.122)	1.07 (1.02–1.13)	0.009 (0.022)
PC/Endothelial cell	0.98 (0.96–1.01)	0.150 (0.167)	0.99 (0.97–1.02)	0.463 (0.463)
EMEC/Endothelial cell	0.99 (0.97–1.01)	0.167 (0.167)	0.99 (0.97–1.01)	0.415 (0.463)

**Table 2:** Univariate and multivariate results in Cox proportional hazards analysis of immune factors in the training cohort. The *p* value was derived from the Cox regression model; FDR-adjusted, multiple-comparison-adjusted *p* values calculated by false discovery rate approach.

	Univariate Analysis		Multivariate Analysis	
**Immune Factors**	**HR (95% CI for HR)**	***p*** **Value (FDR-Adjusted)**	**HR (95% CI for HR)**	***p*** **Value (FDR-Adjusted)**
T adaptive/Monocytes	0.86 (0.78–0.96)	0.004 (0.056)	0.88 (0.80–0.97)	0.010 (0.140)
Monocytes/T adaptive	2.54 (1.24–5.21)	0.011 (0.077)	2.61 (1.11–6.15)	0.028 (0.196)
Monocytes/B adaptive	1.14 (1.00–1.29)	0.048 (0.224)	1.08 (0.95–1.22)	0.239 (0.837)
B adaptive/Monocytes	0.80 (0.61–1.04)	0.097 (0.291)	0.78 (0.59–1.03)	0.078 (0.364)
T Innate/T adaptive	2.76 (0.81–9.39)	0.104 (0.291)	1.60 (0.41–6.21)	0.500 (0.875)
Granulocytes/Monocytes	0.79 (0.50–1.24)	0.304 (0.596)	0.90 (0.63–1.29)	0.582 (0.905)
B adaptive/T Innate	0.99 (0.96–1.02)	0.347 (0.596)	0.99 (0.97–1.01)	0.354 (0.875)
T Innate/B adaptive	1.16 (0.85–1.59)	0.360 (0.596)	1.00 (0.72–1.37)	0.978 (0.997)
B adaptive/T adaptive	1.44 (0.61–3.43)	0.405 (0.596)	1.43 (0.54–3.78)	0.471 (0.875)
Granulocytes/B adaptive	0.87 (0.63–1.22)	0.426 (0.596)	1.05 (0.76–1.45)	0.781 (0.953)
Granulocytes/T Innate	0.96 (0.86–1.09)	0.553 (0.698)	1.00 (0.99–1.01)	0.997 (0.997)
T Innate/Monocytes	0.89 (0.57–1.38)	0.598 (0.698)	0.82 (0.52–1.30)	0.393 (0.875)
T adaptive/B adaptive	0.99 (0.93–1.05)	0.742 (0.749)	0.99 (0.93–1.06)	0.817 (0.953)
Granulocytes/T adaptive	0.85 (0.32–2.29)	0.749 (0.749)	1.17 (0.46–2.98)	0.741 (0.953)

**Table 3:** Multivariate Cox regression analysis used to evaluate the independent risk factor of prognosis in the training cohort.

Variable	HR (95% CI for HR)	*p* Value
STEM score	0.90 (0.84–0.97)	0.003
Age	1.02 (1.00–1.03)	0.019
Gender (Male vs. Female)	1.21 (0.85–1.71)	0.283
Stage (II vs. I)	1.78 (0.68–4.63)	0.239
Stage (III vs. I)	3.58 (1.40–9.15)	0.008
Stage (IV vs. I)	8.49 (3.36–21.47)	<0.001
Lauren (Intestinal vs. Diffuse/Mixed)	0.75 (0.53–1.07)	0.111
Chemotherapy (Yes vs. No)	0.45 (0.31–0.64)	<0.001

**Table 4:** Multivariate Cox regression analysis used to examine the independent risk factor of prognosis in three independent validation cohorts.

	TCGA-STAD		GSE15459		GSE84437	
**Variable**	**HR (95% CI)**	***p*** **Value**	**HR (95% CI)**	***p*** **Value**	**HR (95% CI)**	***p*** **Value**
STEM score	0.94 (0.91–0.97)	0.001	0.89 (0.82–0.97)	0.010	0.86 (0.80–0.92)	<0.001
Age	1.02 (1.00–1.04)	0.011	1.01 (1.00–1.03)	0.128	1.02 (1.01–1.03)	<0.001
Gender (Male vs. Female)	1.09 (0.76–1.57)	0.626	0.74 (0.47–1.19)	0.213	1.31 (0.96–1.77)	0.086
Stage (II vs. I)	1.43 (0.70–2.92)	0.321	2.22 (0.68–7.22)	0.186		
Stage (III vs. I)	2.64 (1.34–5.21)	0.005	7.96 (2.80–22.61)	<0.001		
Stage (IV vs. I)	4.15 (1.89–9.08)	<0.001	23.28 (7.92–68.46)	<0.001		
Lauren (Intestinal vs. Diffuse/Mixed)			1.25 (0.80–1.95)	0.322		
Radiationtherapy (Yes vs. No)	0.41 (0.25–0.69)	0.001				

**Table 5:** Overview of prognostic gene signatures in stromal cell and EMEC subtypes. HR, hazard ratio; CI, confidence interval; DE, differential expression; Up (down), up- (down-) regulated in the TMEsubtype-H compared with TMEsubtype-M and TMEsubtype-L.

Cell Population	Gene Symbol	Gene Name	HR (95% CI)	DE
Stromal cell	FERMT2	fermitin family member 2	1.49 (1.35–1.65)	Up
Stromal cell	SGCE	sarcoglycan epsilon	1.50 (1.35–1.67)	Up
Stromal cell	PPP1R14A	protein phosphatase 1 regulatory inhibitor subunit 14A	1.38 (1.27–1.5)	Up
Stromal cell	LAMC1	laminin subunit gamma 1	1.83 (1.56–2.16)	Up
Stromal cell	MYL9	myosin light chain 9	1.30 (1.21–1.40)	Up
Stromal cell	TPM2	tropomyosin 2	1.34 (1.24–1.46)	Up
Stromal cell	TAGLN	transgelin	1.33 (1.23–1.44)	Up
Stromal cell	AKAP12	A–kinase anchoring protein 12	1.40 (1.27–1.54)	Up
EMEC	KCNQ1	potassium voltage–gated channel subfamily Q member 1	0.73 (0.65–0.82)	Down
EMEC	SURF6	surfeit 6	0.57 (0.45–0.72)	Down
EMEC	AGMAT	agmatinase	0.79 (0.70–0.88)	Down
EMEC	MRPS2	mitochondrial ribosomal protein S2	0.60 (0.48–0.76)	Down

## Data Availability

The data analyzed in this study are available from the Gene Expression Omnibus (accession numbers: GSE62254; GSE15459; GSE84437; GSE134520), The Cancer Genome Atlas Project, or from the authors upon reasonable request.

## Supplementary Materials

The following are available at https://www.mdpi.com/article/10.3390/cancers13215382/s1, Figure S1: Cluster tree of non-immune cell types of patients with NAG, CAG, IM and EGC (Top 50 genes), Figure S2: Cluster tree of non-immune cell types of patients with NAG, CAG, IM and EGC (Top 150 genes), Figure S3: Cluster tree of non-immune cell types of patients with NAG, CAG, IM and EGC (Top 200 genes), Figure S4: Top GO terms enriched for the EMEC gene signatures in the BP (**a**) and CC (**b**) ontologies, Figure S5: Complementary prognostic value of the TME subtypes to the ACRG molecular subtypes, Figure S6: Complementary prognostic value of the TME subtypes to the TCGA genomic subtypes, Figure S7: Forest plot of estimated hazard ratios of prognostic gene signatures in stromal cell subtype, Figure S8: Forest plot of estimated hazard ratios of prognostic gene signatures in EMEC population, Table S1: Clinicopathologic and treatment information for patients in the discovery and validation cohorts, Table S2: Grouping of the non-immune cell types for bulk gene expression samples deconvolution, Table S3: Comparision of deconvolution accuracy on artificial bulk data by combining different signature matrices, Table S4: Grouping of the immune cell types for bulk gene expression samples deconvolution.

## Abbreviations

GC	gastric cancer
TME	tumor microenvironment
EMEC	early malignant epithelial cell
PMEC	premalignant epithelial cell
STEM score	TME signature score by integrating stromal cells, adaptive T cells, EMEC, and monocytes
ACRG	The Asian Cancer Research Group
STAD	stomach adenocarcinoma
EMT	epithelial-to-mesenchymal transition
EBV	Epstein–Barr virus-positivity
MSI	microsatellite instability
GS	genomically stable
CIN	chromosomal instability
TCGA	The Cancer Genome Atlas
CAF	cancer-associated fibroblasts
TIL	tumor-infiltrating lymphocytes
scRNAseq	single-cell RNA-sequencing
NAG	non-atrophic gastritis
CAG	chronic atrophic gastritis
IM	intestinal metaplasia
EGC	early gastric cancer
MSC	metaplastic stem-like cell
PC	proliferative cell
PMC	Pit mucous cell
EC	endothelial cell
GMC	antral basal gland mucous cell
SM cell	smooth muscle cell
TSR	tumor-to-stroma ratio
LMR	lymphocyte-to-monocyte ratio
TPM	transcripts per kilobase million
GO	gene ontology
BP	biological process
CC	cellular component
OS	overall survival
HR	hazard ratio
CI	confidence interval
BH	Benjamini–Hochberg
FDR	false discovery rate.
