# Supplementary material for: The Epithelial and Stromal Immune Microenvironment in Gastric Cancer: A Comprehensive Analysis Reveals Prognostic Factors with Digital Cytometry
Source: Cancers (Basel). 2021 Oct 27;13(21):5382. doi: 10.3390/cancers13215382 (PMC8582557; doi:10.3390/cancers13215382)
Supplement: Supplementary file 1 [file cancers-13-05382-s001.zip › cancers-1384591-supplementary.pdf]

# Supplementary Materials: The Epithelial and Stromal Immune Microenvironment in Gastric Cancer: A Comprehensive Analysis Reveals Prognostic Factors with Digital Cytometry

**Wenjun Shen[1,2,3,*], Guoyun Wang[1], Georgia R. Cooper[4], Yuming Jiang[5,*] and Xin Zhou[4,6]**

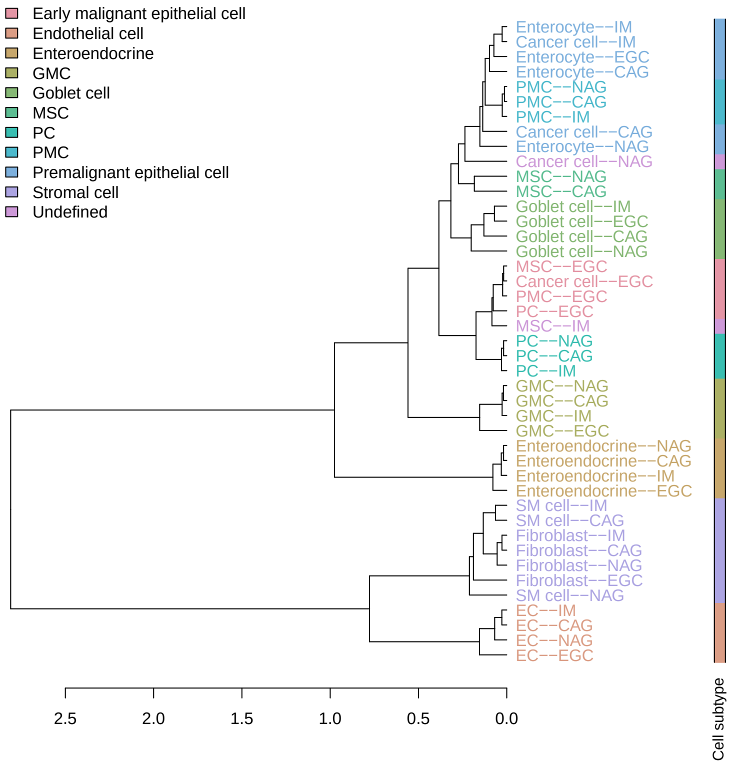


**Figure S1.** Cluster tree of non-immune cell types of patients with NAG, CAG, IM and EGC (Top 50 genes).

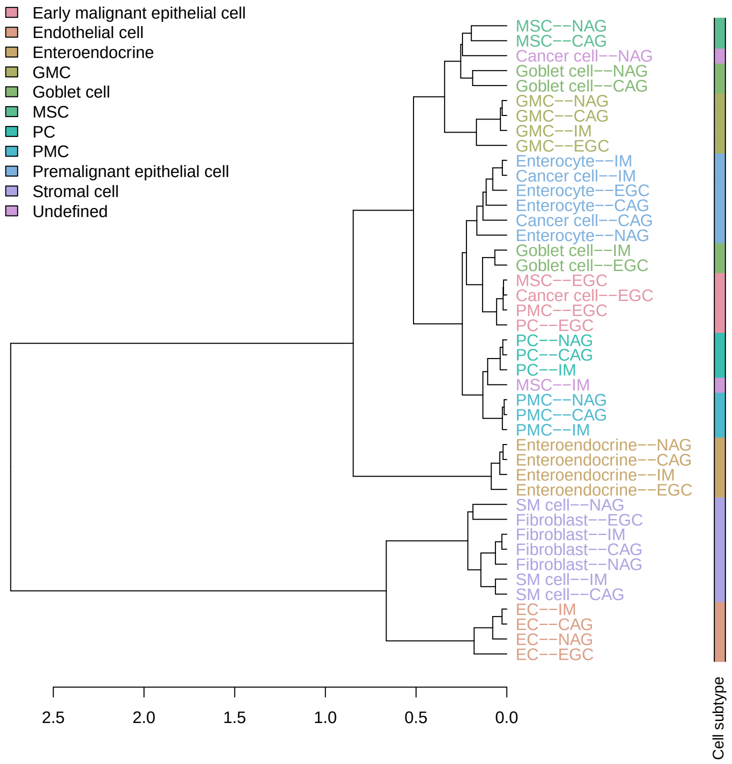


**Figure S2.** Cluster tree of non-immune cell types of patients with NAG, CAG, IM and EGC (Top 150 genes).

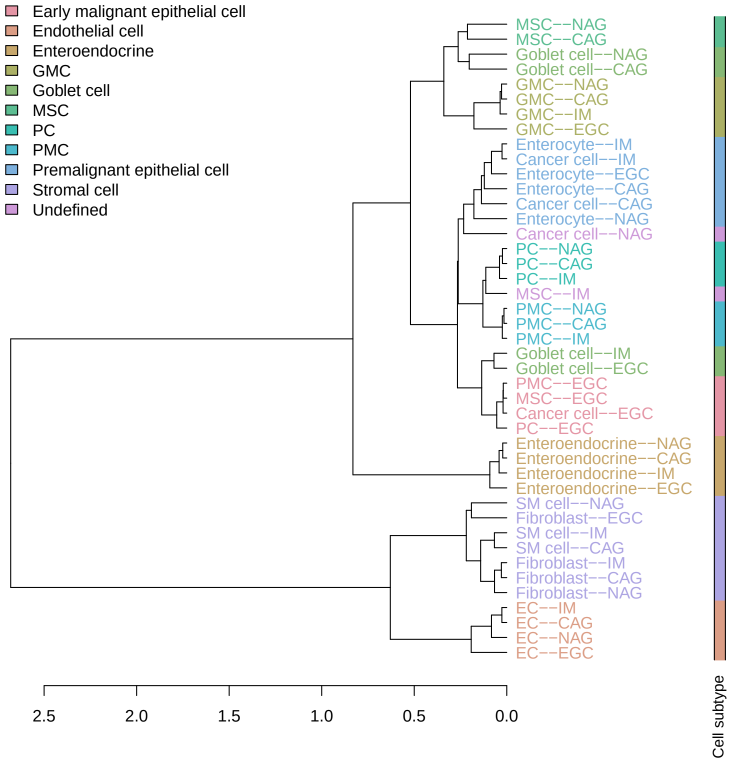


**Figure S3.** Cluster tree of non-immune cell types of patients with NAG, CAG, IM and EGC (Top 200 genes).

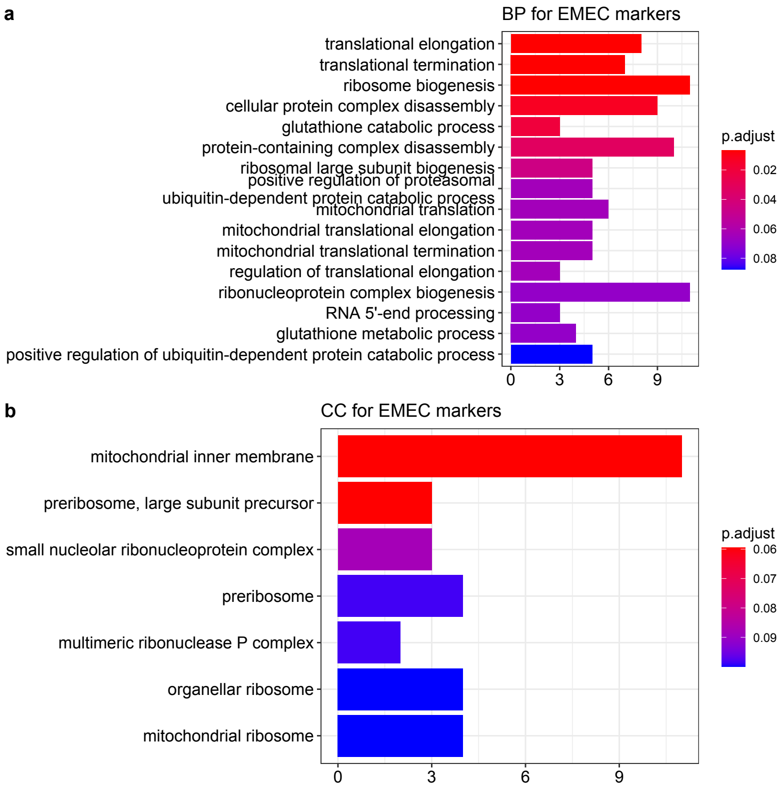


**Figure S4.** Top GO terms enriched for the EMEC gene signatures in the BP (**a**) and CC (**b**) ontologies.

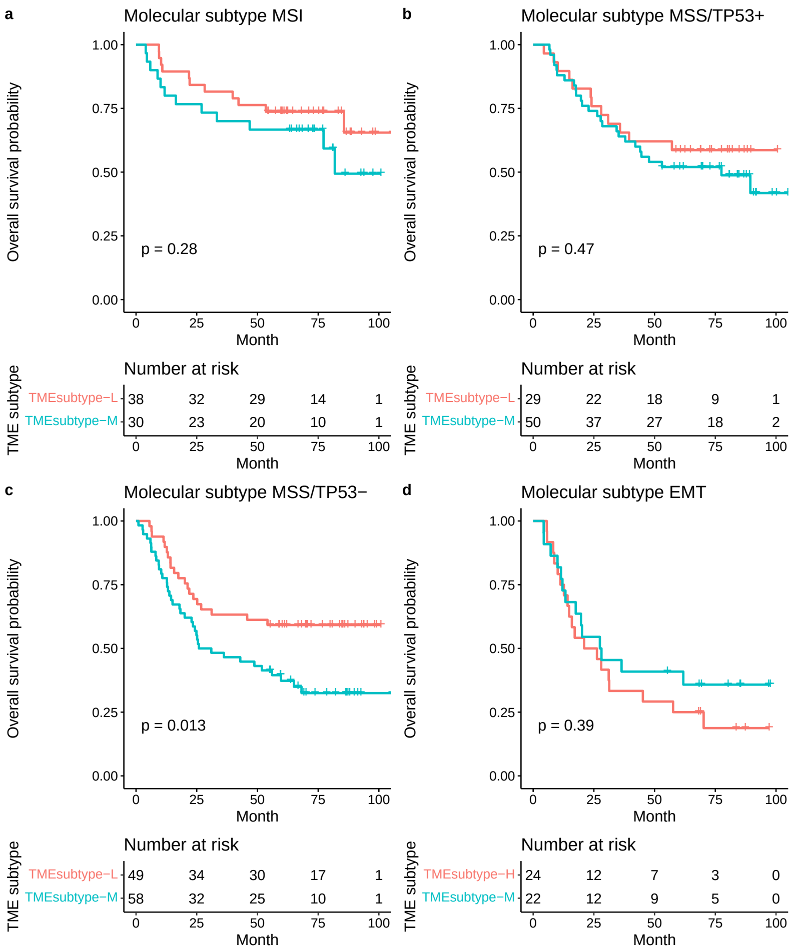


**Figure S5.** Complementary prognostic value of the TME subtypes to the ACRG molecular subtypes.

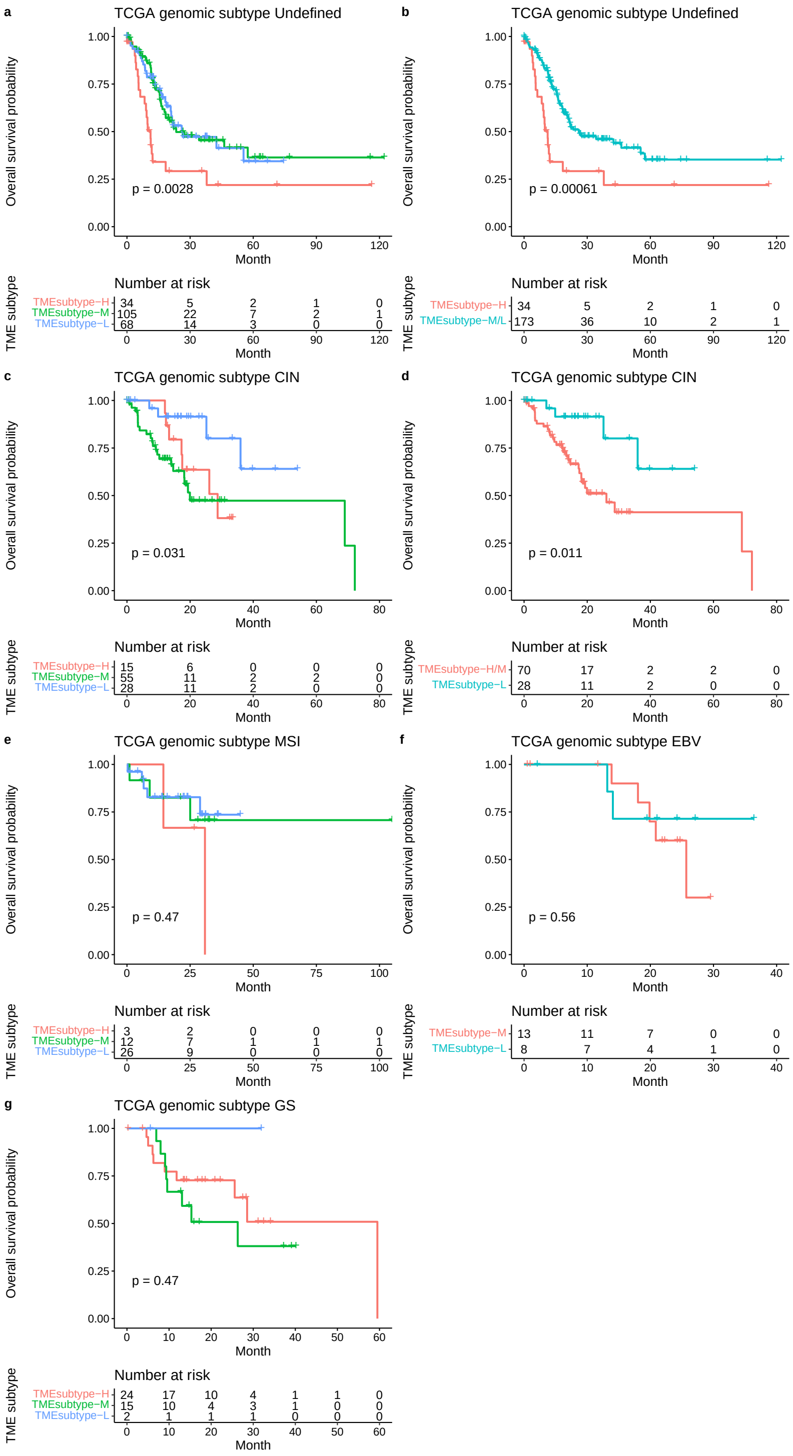


**Figure S6.** Complementary prognostic value of the TME subtypes to the TCGA genomic subtypes.

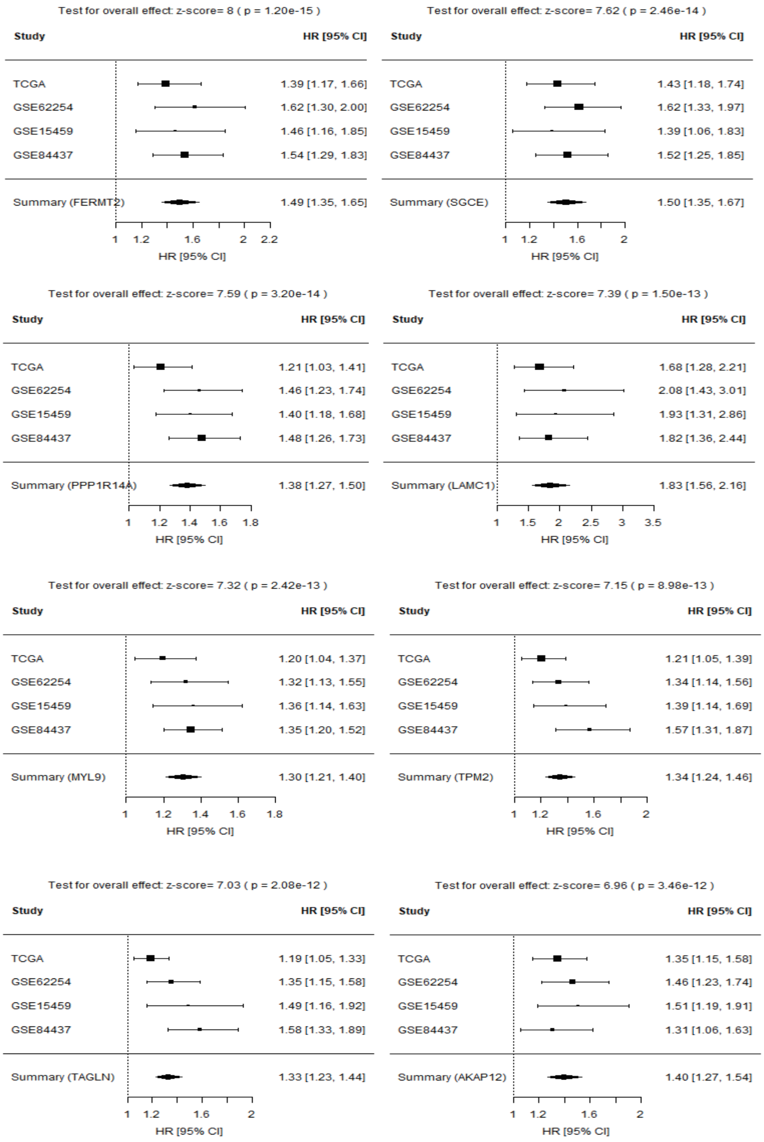


**Figure S7.** Forest plot of estimated hazard ratios of prognostic gene signatures in stromal cell subtype.

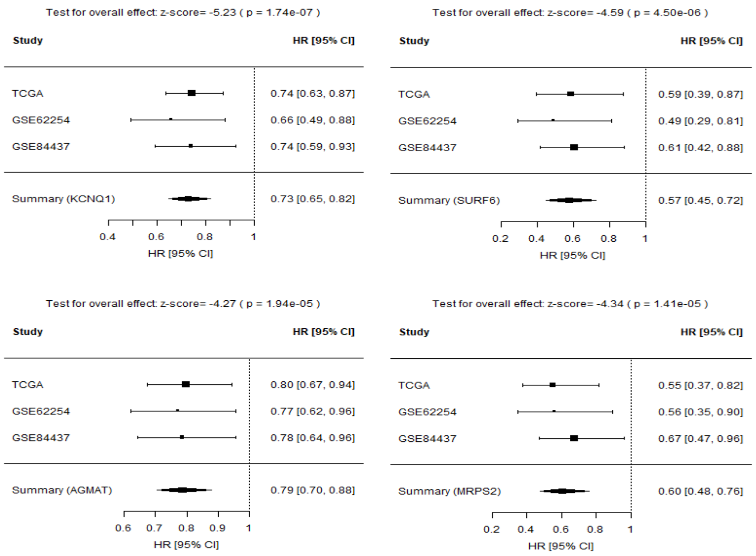


**Figure S8.** Forest plot of estimated hazard ratios of prognostic gene signatures in EMEC population.

**Table S1.** Clinicopathologic and treatment information for patients in the discovery and validation cohorts. A dash indicates that data are not available.

| | ACRG | GSE84437 | GSE15459 | TCGA-STAD |
|---|---|---|---|---|
| No. of patients | 300 | 433 | 192 | 415 |
| Median age in yrs (range) | 64 (24–86) | 62 (27–86) | 67 (23–92) | 67 (30–90) |
| Male (%) | 199 (66) | 296 (68) | 125 (65) | 268 (65) |
| Stage | | | | |
| I | 30 (10) | - | 31 (16) | 57 (15) |
| II | 96 (32) | - | 29 (15) | 123 (32) |
| III | 95 (32) | - | 72 (38) | 169 (43) |
| IV | 77 (26) | - | 60 (31) | 41 (11) |
| Lauren (%) | | | | |
| Intestinal | 146 (49) | - | 99 (52) | 176 (72) |
| Diffuse/mixed | 151 (51) | - | 93 (48) | 69 (28) |
| Chemotherapy (%) | 144 (48) | - | - | - |
| Radiationtherapy | - | - | - | 72 (19) |
| Median follow-up (mo.) | 80 | 116 | 74 | 22 |

**Table S2.** Grouping of the non-immune cell types for bulk gene expression samples deconvolution.

| Non-immune cell types | Full name | Stages | Grouping for Bulk deconvolution |
|---|---|---|---|
| Cancer cell | Cancer cell | EGC | EMEC |
| MSC | Metaplastic stem-like cell | EGC | EMEC |
| PC | Proliferative cell | EGC | EMEC |
| PMC | Pit mucous cell | EGC | EMEC |
| EC | Endothelial cell | CAG | Endothelial cell |
| EC | Endothelial cell | EGC | Endothelial cell |
| EC | Endothelial cell | IM | Endothelial cell |
| EC | Endothelial cell | NAG | Endothelial cell |
| Enteroendocrine | Enteroendocrine cell | CAG | Enteroendocrine |
| Enteroendocrine | Enteroendocrine cell | EGC | Enteroendocrine |
| Enteroendocrine | Enteroendocrine cell | IM | Enteroendocrine |
| Enteroendocrine | Enteroendocrine cell | NAG | Enteroendocrine |
| GMC | Antral basal gland mucous cell | CAG | GMC |
| GMC | Antral basal gland mucous cell | EGC | GMC |
| GMC | Antral basal gland mucous cell | IM | GMC |
| GMC | Antral basal gland mucous cell | NAG | GMC |
| Goblet cell | Goblet cell | CAG | Goblet cell |
| Goblet cell | Goblet cell | EGC | Goblet cell |
| Goblet cell | Goblet cell | IM | Goblet cell |
| Goblet cell | Goblet cell | NAG | Goblet cell |
| MSC | Metaplastic stem-like cell | CAG | MSC |
| MSC | Metaplastic stem-like cell | NAG | MSC |
| PC | Proliferative cell | CAG | PC |
| PC | Proliferative cell | IM | PC |
| PC | Proliferative cell | NAG | PC |
| PMC | Pit mucous cell | CAG | PMC |
| PMC | Pit mucous cell | IM | PMC |
| PMC | Pit mucous cell | NAG | PMC |
| Cancer cell | Cancer cell | CAG | PMEC |
| Cancer cell | Cancer cell | IM | PMEC |
| Enterocyte | Enterocyte | CAG | PMEC |
| Enterocyte | Enterocyte | EGC | PMEC |
| Enterocyte | Enterocyte | IM | PMEC |
| Enterocyte | Enterocyte | NAG | PMEC |
| Fibroblast | Fibroblast | CAG | Stromal cell |
| Fibroblast | Fibroblast | EGC | Stromal cell |
| Fibroblast | Fibroblast | IM | Stromal cell |
| Fibroblast | Fibroblast | NAG | Stromal cell |
| SM cell | Smooth muscle cell | CAG | Stromal cell |
| SM cell | Smooth muscle cell | IM | Stromal cell |
| SM cell | Smooth muscle cell | NAG | Stromal cell |
| Cancer cell | Cancer cell | NAG | Undefined |
| MSC | Metaplastic stem-like cell | IM | Undefined |

**Table S3.** Comparision of deconvolution accuracy on artificial bulk data by combining different signature matrices. The table shows average root mean square error (RMSD), mean absolute deviation (mAD), and Pearson correlation (R) across all samples and cell types.

| Top M genes | RMSD | mAD | R |
|---|---|---|---|
| Top 50 | 0.0837 | 0.0492 | 0.8907 |
| Top 100 | 0.0837 | 0.0492 | 0.8907 |
| Top 150 | 0.0836 | 0.0488 | 0.8923 |
| Top 200 | 0.0840 | 0.0488 | 0.8917 |
| Top 250 | 0.0847 | 0.0495 | 0.8899 |

**Table S4.** Grouping of the immune cell types for bulk gene expression samples deconvolution.

| Immune cell types | Full name | Grouping for Bulk deconvolution |
|---|---|---|
| Monocytes C | Classical monocytes | Monocytes |
| Monocytes NC+I | Non-classical/intermediate monocytes | Monocytes |
| NK | Natural killer cells | NK |
| T gd non-Vd2 | $\gamma\delta$ non-V$\delta$2 T cells | T Innate |
| T gd Vd2 | $\gamma\delta$ V$\delta$2 T cells | T Innate |
| Neutrophils LD | Low-density neutrophils | Granulocytes |
| Basophils LD | Low-density basophils | Granulocytes |
| mDCs | Myeloid dendritic cells | DCs |
| pDCs | Plasmacytoid dendritic cells | DCs |
| T CD8 Naive | Naïve CD8 T cells | T adaptive |
| T CD8 Memory | Memory CD8 T cells | T adaptive |
| B Naive | Naive B cells | B adaptive |
| B Memory | Memory B cells | B adaptive |
| Plasmablasts | Plasmablasts | B adaptive |
| T CD4 Naive | Naïve CD4 T cells | T adaptive |
| T CD4 Memory | Memory CD4 T cells | T adaptive |
| MAIT | Mucosal associated invariant T cells | T Innate |
